# Mechanistic Insights on the In Vitro Antibacterial Activity and In Vivo Hepatoprotective Effects of *Salvinia auriculata* Aubl against Methotrexate-Induced Liver Injury

**DOI:** 10.3390/ph15050549

**Published:** 2022-04-29

**Authors:** Nashwah G. M. Attallah, Fatma Alzahraa Mokhtar, Engy Elekhnawy, Selim Z. Heneidy, Eman Ahmed, Sameh Magdeldin, Walaa A. Negm, Aya H. El-Kadem

**Affiliations:** 1 Department of Pharmaceutical Science, College of Pharmacy, Princess Nourah Bint Abdulrahman University, P.O. Box 84428, Riyadh 11671, Saudi Arabia; ngmohamed@pnu.edu.sa; 2 Department of Pharmacognosy, Faculty of Pharmacy, ALSalam University, Kafr El Zayat 31616, Al Gharbiya, Egypt; 3 Pharmaceutical Microbiology Department, Faculty of Pharmacy, Tanta University, Tanta 31527, Egypt; engy.ali@pharm.tanta.edu.eg; 4 Department of Botany & Microbiology, Faculty of Science, Alexandria University, Alexandria 21521, Egypt; selim.heneidy@alexu.edu.eg; 5 Department of Pharmacology, Faculty of Veterinary Medicine, Suez Canal University, Ismailia 41522, Egypt; eman.abdelnaby@57357.org; 6 Proteomics and Metabolomics Research Program, Department of Basic Research, Children’s Cancer Hospital 57357, Cairo 11441, Egypt; sameh.magdeldin@57357.org; 7 Department of Physiology, Faculty of Veterinary Medicine, Suez Canal University, Ismailia 41522, Egypt; 8 Department of Pharmacognosy, Faculty of Pharmacy, Tanta University, Tanta 31527, Egypt; 9 Department of Pharmacology and Toxicology, Faculty of Pharmacy, Tanta University, Tanta 31527, Egypt; aya.elkadeem@pharm.tanta.edu.eg

**Keywords:** *Acinetobacter baumannii*, inflammasome, LC-ESI-MS/MS, NLPR3, oxidative stress, SEM

## Abstract

Methotrexate (MTX) is widely used in the treatment of numerous malignancies; however, its use is associated with marked hepatotoxicity. Herein, we assessed the possible hepatoprotective effects of *Salvinia auriculata* methanol extract (SAME) against MTX-induced hepatotoxicity and elucidated the possible fundamental mechanisms that mediated such protective effects for the first time. Forty mice were randomly allocated into five groups (eight/group). Control saline, MTX, and MTX groups were pre-treated with SAME 10, 20, and 30 mg/kg. The results revealed that MTX caused a considerable increase in blood transaminase and lactate dehydrogenase levels, oxidative stress, significant activation of the Nod-like receptor-3 (NLPR3)/caspase-1 inflammasome axis, and its downstream inflammatory cytokines interleukin-1β (IL-1β) and interleukin-18 (IL-18). MTX also down-regulated nuclear factor erythroid 2-related factor 2 (Nrf2) expression. Additionally, it increased the immunostaining of nuclear factor kappa-B (NF-κB) and downstream inflammatory mediators. Furthermore, the hepatic cellular apoptosis was dramatically up-regulated in the MTX group. On the contrary, prior treatment with SAME significantly improved biochemical, histopathological, immunohistochemical alterations caused by MTX in a dose-dependent manner. The antibacterial activity of SAME has also been investigated against *Acinetobacter baumannii* clinical isolates. LC-ESI-MS/MS contributed to the authentication of the studied plant and identified 24 active constituents that can be accountable for the SAME-exhibited effects. Thus, our findings reveal new evidence of the hepatoprotective and antibacterial properties of SAME that need further future investigation.

## 1. Introduction

Acute hepatic damage may progress to chronic hepatitis or even liver failure, inducing high mortality and morbidity worldwide. Various inducers may trigger it, including alcohol, drugs, viral infections, metabolic and autoimmune diseases [1]. Methotrexate (MTX) is commonly used to address a wide variety of ailments, including cancer, multiple sclerosis, psoriasis, rheumatoid arthritis, and different inflammatory disorders [2]. MTX cytotoxicity is not confined to the malignant cells only, but also affects normal cells of different organs. Hence, the extended use of MTX for a long time has been related to multiple-organ toxicity. MTX hepatotoxicity puts a great limitation on its use in the labeled doses. Therefore, natural products attract attention nowadays since they are safe, effective, and lack side effects.

The floating aquatic fern *Salvinia auriculata* Aubl. flourishes in slow-moving, nutrient-rich, and warm freshwater. It is a competitive and fast-growing plant that may be found all over the tropics and subtropics. *S. auriculata* can proliferate within days, forming floating dense mats, even in oligotrophic environments [3], which decrease the flow of water and the amount of light and oxygen in the water. It is marketed as an aquatic ornamental plant. It can be used to eliminate lead and other heavy metal pollutants from wastewater in artificial wetlands [4,5,6]. *S. auriculata* can be utilized as a mulch because of its quick growth and nutrient uptake. It has also been proposed as a bioindicator in aquatic habitats, where it might be used to monitor cadmium-contaminated water [6]. *S. auriculata* can be used for medicinal purposes as the different species are interesting natural antioxidant sources and could be considered a potential source of valuable drugs [7,8,9,10]. Given the lack of adequate research on *S. auriculata’s* biological and pharmacological effects, we conducted this study.

Many bacterial infections are associated with liver diseases, and *Acinetobacter* species are commonly isolated from patients with acute and chronic liver injury [11]. *Acinetobacter baumannii* is already one of the most common opportunistic pathogenic bacteria in clinical settings. Unfortunately, these bacteria acquire many resistance genes to many antibiotics, increasing the bacteria’s ability to survive and spread [12]. In addition, the virulence factors of these bacteria, especially their ability to form a biofilm, are an important characteristic of *A. baumannii* that allows them to escape the immune system and harsh environments [13]. Thus, more efforts are required to find new therapeutic alternatives to fight this pathogen.

Different reports had demonstrated the essential role of the oxidative stress, inflammatory cytokines, and involvement of nuclear factor kappa-B (NF-κB) pathway in mitigating the hepatotoxic effects of MTX [14,15,16]. However, the exact underlying mechanism of MTX hepatotoxicity is not clearly defined. Thus, our research aims to evaluate the possible protective consequences of *Salvinia auriculata* methanolic extract (SAME) against MTX-induced hepatotoxicity for the first time, in addition to revealing the possible underlying mechanisms of such protective factors’ effects. Moreover, phytochemical profiling of SAME and its in vitro antibacterial activity against *A. baumannii* were investigated. Finally, we summarized the work in the graphical abstract.

## 2. Results

### 2.1. LC-ESI-MS/MS Analysis

LC-MS/MS identified the metabolites in SAME. Using positive and negative ion modes of LC-MS/MS, 24 metabolites were tentatively recognized. The main compounds are divided into several subclasses: coumarins, flavonoids, phenolic, alkaloid, and organic acids. Comprehensive profiling was presented in Table 1, and the total ion chromatogram (TIC) of SAME was displayed in supplementary Figure S1.

### 2.2. Characterization of the Identified Compounds

**Figure 1:**
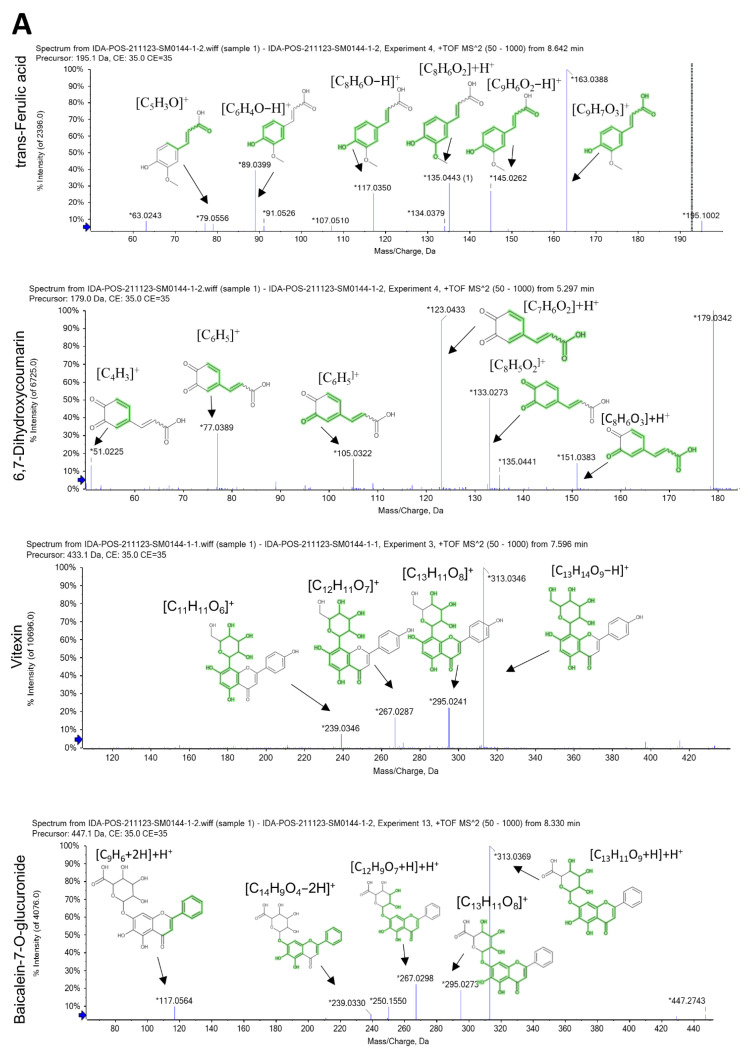

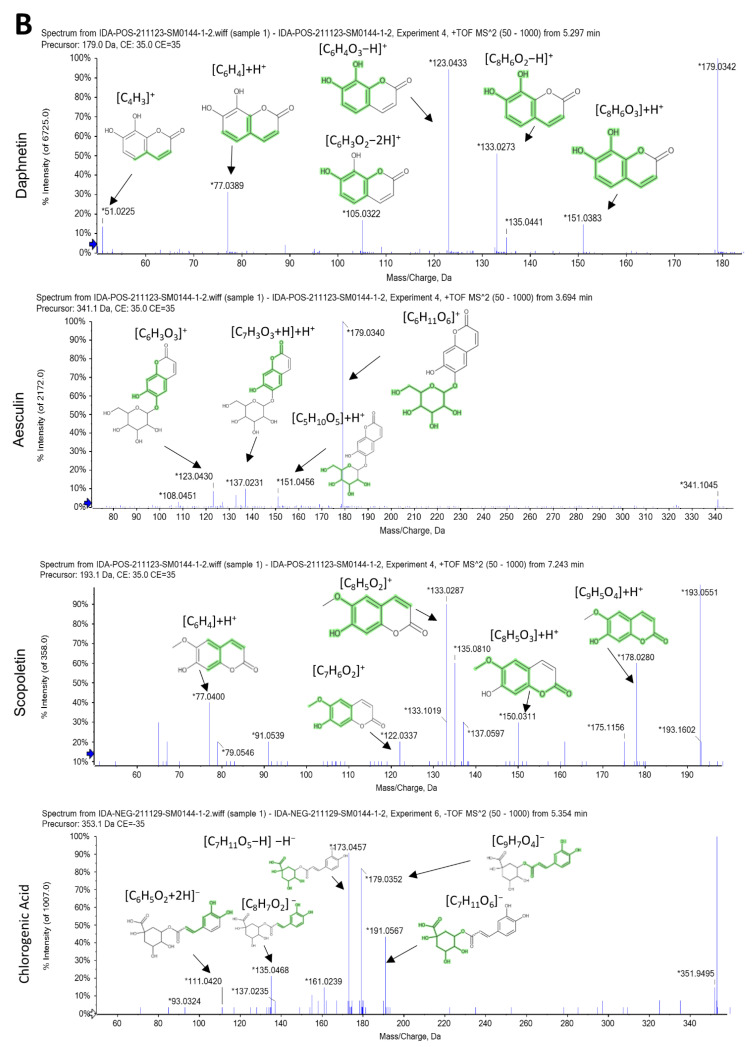
Mass/Mass spectra showing fragmentation pattern of major identified compounds. (**A**) *Trans*-ferulic acid, 6,7-dihydroxy coumarin vitexin, and baicalein-7-*O*-glucuronide. (**B**) Daphnetin, esculin, scopoletin, and chlorogenic acid. * mean precursor ion (*m*/*z*).

Coumarins are the major detected compounds in SAME. Of these, 6,7-dihydroxy coumarin (*m*/*z* 179.033 [M+H] ^+^) is predominant in both positive and negative ion modes (Figure 1A), while daphnetin (*m*/*z* 179.033), esculin (*m*/*z* 341.08) and scopoletin (*m*/*z* 193.04) are present in positive ion modes (Figure 1B).

Only one xanthine alkaloid (caffeine) is detected in positive mode, displaying characteristic MS/MS fragments at *m*/*z* 110.071 [C_5_H_7_N_3_]+H^+^, 138.066 [C_6_H_7_N_3_O]+H^+^, 151.037 [C_6_H_4_N_3_O_2_]+H^+^.

According to our LC-MS/MS analysis, SAME exhibited a variety of organic or phenolic compounds such as caffeic acid (*m*/*z* 179.04 [M-H]^−^), chlorogenic acid in positive and negative ion modes, 3-hydroxyanthranilic acid, *trans*-ferulic acid, and jasmonic acid (*m*/*z* 154.04, 195.06, 211.13, respectively) in positive modes, with *trans*-cinnamate (*m*/*z* 147.04) present in negative ion modes. Jasmonic acid is reported for the first time in *Salvinia* spp.

*Trans*-ferulic acid is the major phenolic acid in SAME, exhibiting characteristic MS/MS fragments at *m*/*z* 63.02 [C_5_H_4_−H]^+^, 79.05 [C_5_H_3_O]^+^, 117.03 [C_8_H_6_O−H]^+^, 135.04 [C_8_H_6_O_2_]+H^+^, 145.02 [C_9_H_6_O_2_−H]^+^, 163.03 [C_9_H_7_O_3_]^+^ (Figure 1A) followed by chlorogenic acid that displayed characteristic MS/MS fragments at *m*/*z* 111.04 [C_6_H_5_O_2_+2H]^−^, 135.04 [C_8_H_7_O_2_]^−^, 161.02 [C_9_H_7_O_3_-H]−H^−^, 173.04 [C_7_H_11_O_5_−H]−H^−^, 179.03 [C_9_H_7_O_4_]^−^, and 191.05 [C_7_H_11_O_6_]^−^ (Figure 1B).

The MS/MS fragment [C_7_H_4_O_4_]+H^+^ at *m*/*z* 153.02 is found in luteolin, apigenin, and other flavonoids with 5,7-dihydroxy, while the MS/MS fragment 135.044 [C_8_H_6_O_2_]+H^+^ at *m/z* for 3′,4′-dihydroxy flavones is suggestive for luteolin. This fragment is replaced by the [C_8_H_6_O]+H^+^ fragment at *m*/*z* 119.08 for apigenin. The major detected flavonoid -*C*-glycoside is vitexin (*m*/*z* 433.11), and baicalein-7-*O*-glucuronide (*m*/*z* 447.09) from flavonoid -*O*-glycoside, Cyanidin 4″-glucoside (*m*/*z* 449.10) from anthocyanidin-3-*O*-glycosides in positive mode analysis. While in negative mode, 6-hydroxy kaempferol-3-glucoside (*m*/*z* 463.08) is identified from flavonoid-*O*-glycoside. Besides the fragment ion for ring A, isoflavonoid such as daidzein and genistein have the fragment ion [M+H−CO]^+^. 

### 2.3. In Vitro Antibacterial Activity of SAME

We performed antimicrobial susceptibility testing of 13 antimicrobials against *A. baumannii* clinical isolates (*n* = 27) using the Kirby–Bauer method. The resistance profile of tested *A. baumannii* isolates is presented in Table 2.

SAME exhibited antibacterial activity on the tested *A. baumannii* isolates by the agar well diffusion method. In addition, the minimum inhibitory concentration (MIC) values of SAME were compelled using the broth microdilution method, ranging from 16 to 128 µg/mL as shown in Table S1.

### 2.4. Mechanism of Action of the Antibacterial Activity of SAME

Herein, we investigated the possible different mechanisms of action of SAME as an antibacterial agent.

#### 2.4.1. Cell Membrane Integrity

**Figure 2:**
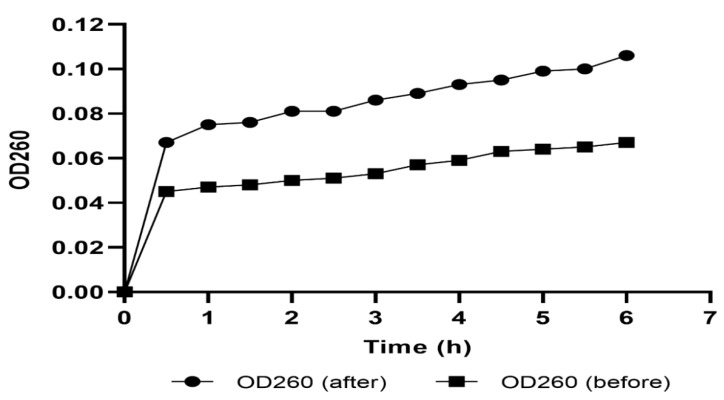
An illustrative example for the significant decrease in the membrane integrity in *A. baumannii* isolate (A1) after treatment with SAME.

The integrity of the membranes of the tested *A. baumannii* isolates was assessed before and after treatment with SAME (at 0.5 MIC values) by detecting the discharge of the material that absorbs at 260 nm. We observed a profound reduction (*p* < 0.05) in the membrane integrity in 51.85% of the treated isolates (Figure 2).

#### 2.4.2. Cell Membrane Permeability

**Figure 3:**
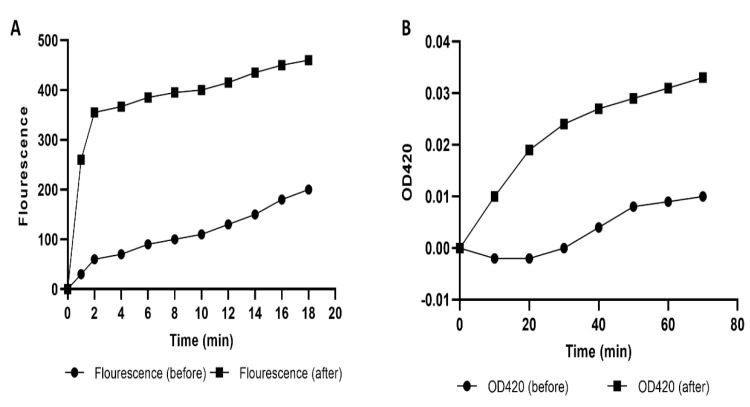
An illustrative example of *A. baumannii* isolate (A1) shows a significant rise in (**A**) the outer membrane permeability and (**B**) the inner membrane permeability after treatment with SAME.

The permeability of the outer membrane was evaluated in the treated isolates compared to the non-treated ones by detecting the fluorescence of N-phenyl-1-naphthylamine (NPN). We noticed a substantial rise (*p* < 0.05) in the permeability of the outer membrane after treatment with SAME (0.5 MIC values) in 55.56% of the isolates (Figure 3A). 

In addition, the permeability of the inner membrane of *A. baumannii* isolates was investigated using *O*-nitrophenyl-*β*-D-galactopyranoside (ONPG), which can enter the bacterial cytoplasm where it is cleaved by the β-galactosidase enzyme. *O*-nitrophenol (ONP) is produced; it has a yellow color which can be monitored by the determination of the absorbance at OD_420_ concerning time. 

We noticed a remarkable increase (*p* < 0.05) in the inner membrane permeability after treatment with SAME (0.5 MIC values) in 59.26% of *A. baumannii* isolates (Figure 3B). 

#### 2.4.3. Protein Content

**Figure 4:**
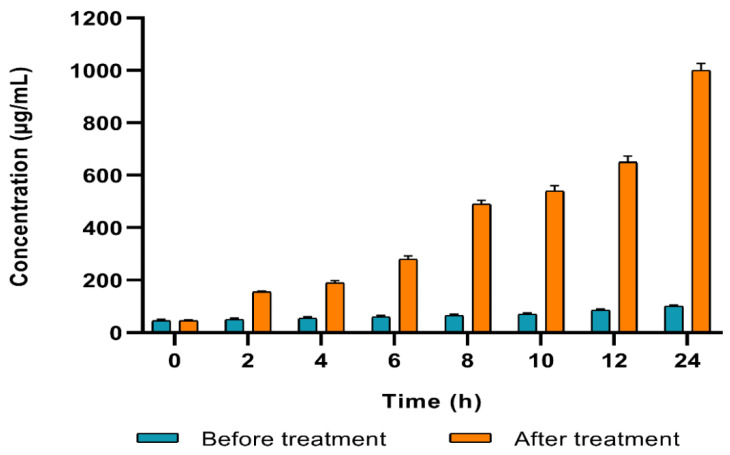
An illustrative example for the isolate (A1), showing a significant rise in the protein concentration after treatment with SAME.

The developed color intensity is directly proportional to protein concentration in the culture medium. We observed a substantial rise (*p* < 0.05) in the protein concentration in 48.15% of *A. baumannii* isolates after treatment with SAME in comparison with the non-treated isolates (Figure 4).

#### 2.4.4. Bacterial Morphology

**Figure 5:**
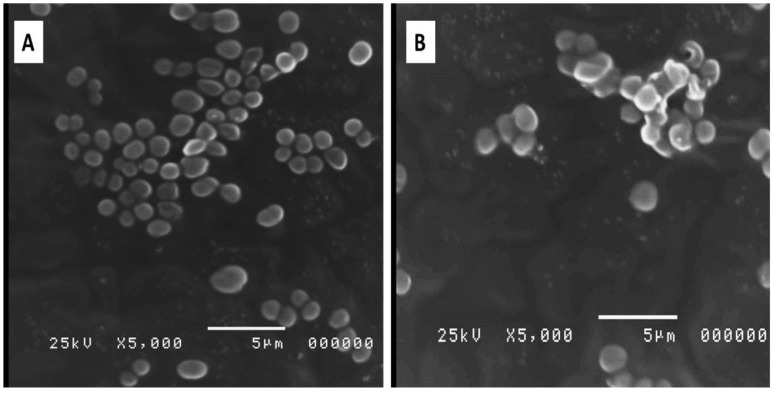
Scanning electron image showing the morphology of a representative *A. baumannii* isolate (A1): (**A**) before and (**B**) after treatment with SAME.

The impact of SAME on the morphology of *A. baumannii* isolates was elucidated using SEM. After treatment with SAME (0.5 MIC values), the cell wall was disrupted, and the cells were lysed, as shown in Figure 5.

### 2.5. In Vivo Hepatoprotecive Activity of SAME

#### 2.5.1. Effects on Serum Indices of Hepatotoxicity

The alteration in serum liver enzymes is an indicative and reliable marker for hepatotoxicity; thus, it is essential to determine the serum level of different liver enzymes. As shown in Table 3, the use of MTX led to a rise in serum levels of alanine amino transferase (ALT), aspartate amino transferase (AST), and lactate dehydrogenase (LDH) (29.33, 122.7, 97.27%, respectively) when compared to the control group. Pretreatment with SAME at different doses induced a significant decrease in AST level at dose level 20 and 30 (13.86, 75.54%, respectively, dose-dependent). Furthermore, it caused a dose-dependent reduction in ALT levels (5.04, 13.07, 20.1%, respectively). LDH levels were significantly reduced by SAME treatment at dose level 20 and 30 (33.13, 44.85%, respectively) in comparison with the MTX group in a dose–response fashion (Table 3).

#### 2.5.2. Effects on Hepatic Oxidative Stress Markers

Oxidative stress is reported to play a dramatic role in the pathophysiology of chronic and acute liver diseases caused by different etiologies. In the MTX group, oxidative damage increased dramatically, as shown in Table 4. MTX induced marked elevation in the hepatic lipid peroxidation manifested by a substantial increase in malondialdhyde (MDA) content (55.4%) compared to the normal group. Furthermore, MTX showed pronounced elevation in hepatic nitric oxide (NO) levels (161.41%) and significantly depressed superoxide dismutase (SOD) activity (28.81%) in the hepatic compared to the normal group. Pretreatment with SAME 20 and 30 alleviated oxidative damage markers and enhanced the liver antioxidant capability. The effects with SAME 10 group were non-significant. Significantly decreased MDA levels (19.56, 31.30% in SAME 20 and 30 groups respectively) were found in a dose-dependent manner compared to the MTX group. SAME 30 could almost completely abrogate MDA elevation. Interestingly, SOD activity was restored to control levels by SAME 30 group treatment (Table 4). Results showed that SAME caused a decrease of about 18.37, 34.93, or 57.22% in NO levels dose-dependently.

#### 2.5.3. Effects on Nod-Like Receptor-3 (NLPR3) Inflammasome Signaling Axis

**Figure 6:**
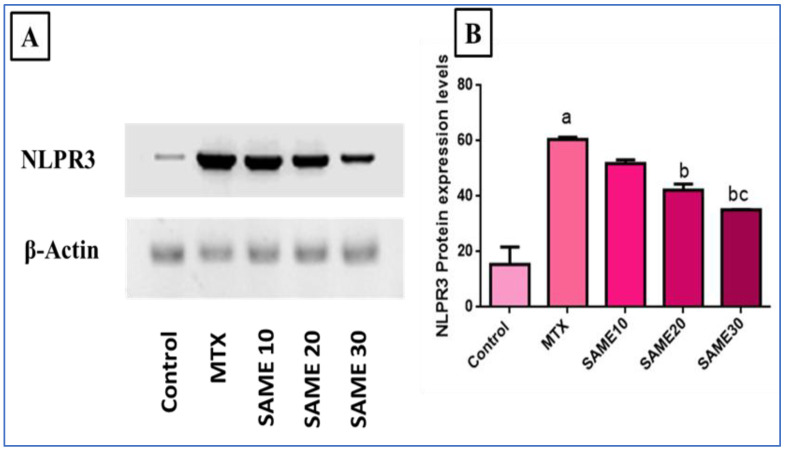
Effect of the SAME pre-treatment on the expression of NLPR3 in the liver tissues. (**A**) Representative western blot bands of NLPR3/β-actin (**B**) Graphical presentation of NLPR3 expression level in hepatic tissue. Hepatotoxicity was induced by a single I.P injection of MTX (20 mg/kg). SAME 10, 20 and 30 were given orally once daily for 5 consecutive days and a single injection of MTX on the sixth day. Data expressed as mean ± SD (*n* = 8/group) as the experiments were performed in three independent triplicates. Significant difference vs. ^a^ respective control, ^b^ respective MTX group, ^c^ respective SAME 10 group, each at *p* < 0.05.

The NLPR3 inflammasome is a significant inflammation signaling pathway triggered by multiple factors, and its activation, in turn, activates its downstream inflammatory and apoptotic markers. In our research, NLPR3 expression was measured by western blot. As evident in Figure 6, MTX markedly up-regulated NLPR3 expression (456.23%) relative to the normal control. Meanwhile, prior use of SAME 10, 20, and 30 powerfully depressed expression levels (13.67, 28.36, 42.74%, respectively) in comparison to the MTX group. The impact was more significant in the SAME 30 group (Supplementary Figure S2).

**Figure 7:**
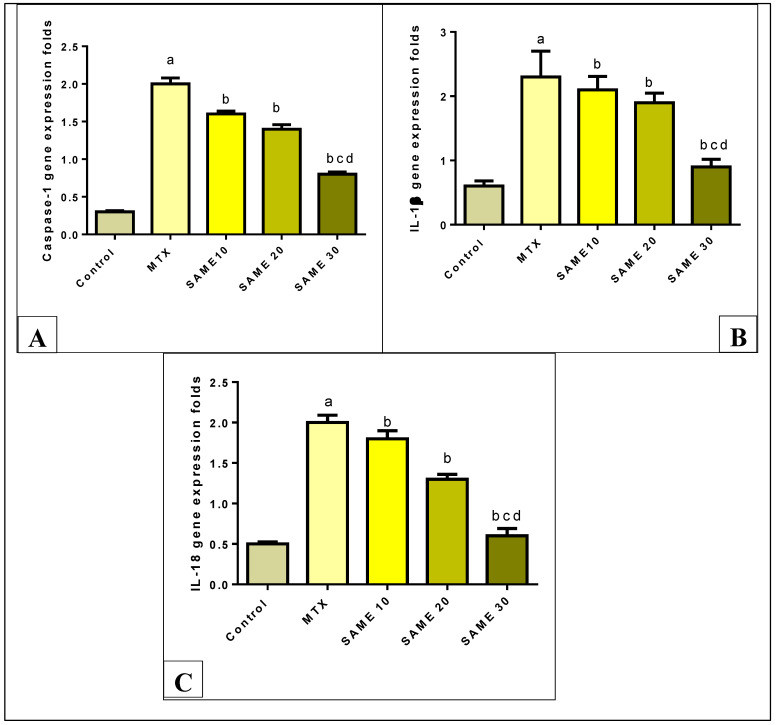
Effect of the SAME pre-treatment on (**A**) Hepatic caspase-1 gene expression level (**B**) Hepatic IL-1β level (**C**) Hepatic IL-18 gene expression level. Hepatotoxicity was induced by a single I.P injection of MTX (20 mg/kg). SAME 10, 20 and 30 were given orally once daily for 5 consecutive days and a single injection of MTX on the sixth day. Data expressed as mean ± SD (*n* = 8/group). Significant difference vs. ^a^ respective control, ^b^ respective MTX group, ^c^ respective SAME 10 group, ^d^ respective SAME 20 group each at *p* < 0.05.

MTX-treated mice displayed substantially higher levels of caspase-1 expression (566.6%) compared to control. Prior therapy with SAME 10, 20, and 30 induced a remarkable decrease in caspase-1 expression levels (20, 30, 60%, respectively) dose-dependently compared to the MTX group (Figure 7A), (*p* < 0.05).

Figure 7B shows that the MTX group demonstrated considerably up-regulated interleukin-1β (IL-1β) expression (283.3%) levels in comparison to the control group. SAME 10, 20, and 30 pre-treated groups substantially suppressed IL-1β expression levels (8.69, 17.39, 60.86%, respectively) in relation to the MTX group, having a superior effect in the SAME 30 group (Figure 7B), (*p* < 0.05).

The present study found that the MTX-intoxicated group had markedly up-regulated expression levels of interleukin-18 (IL-18) (300%) compared to the control group. Pretreatment with SAME 10, 20, and 30 substantially diminished IL-18 expression levels (10, 35, 70%, respectively) compared to the MTX group dose-dependently (Figure 7C), (*p* < 0.05).

#### 2.5.4. Effects on Hepatic Nuclear Factor Erythroid 2-Related Factor 2 (Nrf2) Gene Expression

**Figure 8:**
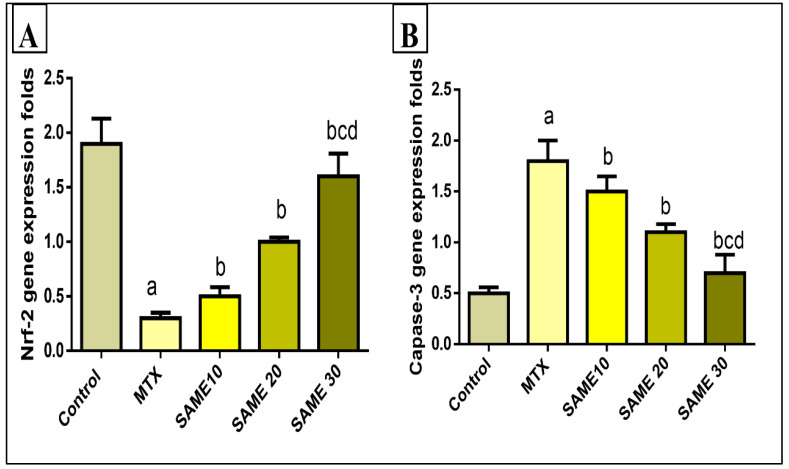
Effect of the SAME pre-treatment on (**A**) Nrf-2 expression level (**B**) Caspase-3 expression level. Hepatotoxicity was induced by a single I.P injection of MTX (20 mg/kg). SAME 10,20 and 30 were given orally once daily for 5 consecutive days and a single injection of MTX on the sixth day. Data expressed as mean ± SD (*n* = 8/group). Significant difference vs. ^a^ respective control, ^b^ respective MTX group, ^c^ respective SAME 10 group, ^d^ respective SAME 20 group each at *p* < 0.05.

Nrf2 is a key endogenous oxidative stress regulator which promotes antioxidant target genes expression and thus induces a variety of beneficial effects in the liver. In the current study, MTX significantly down-regulated Nrf2 expression in the liver tissue (84.21%) compared to the control group. This reduction in Nrf2 expression is an indicator of the lowering of the antioxidant status of the liver. Pretreatment with SAME 10, 20, and 30 up-regulated Nrf2 mRNA expression (66.66, 233.3, 433.3%) dose-dependently compared to the MTX group (Figure 8A).

#### 2.5.5. Effects on the Hepatic Caspase-3 Gene Expression

The role of apoptosis in MTX-induced hepatotoxicity should be explored. Thus, apoptosis markers such as caspase-3 were assessed in the current study. MTX treatment induced a striking up-regulation in liver caspase-3 gene expression (260%) relative to the control group. SAME 10, 20, and 30-treated groups displayed a prominent downregulation in caspase-3 expression (16.66, 38.9, 72.22%, respectively) compared to the MTX group. In the SAME 30 group, the effect is more pronounced (Figure 8B), (*p* < 0.05).

### 2.6. Immunohistochemical Studies

**Figure 9:**
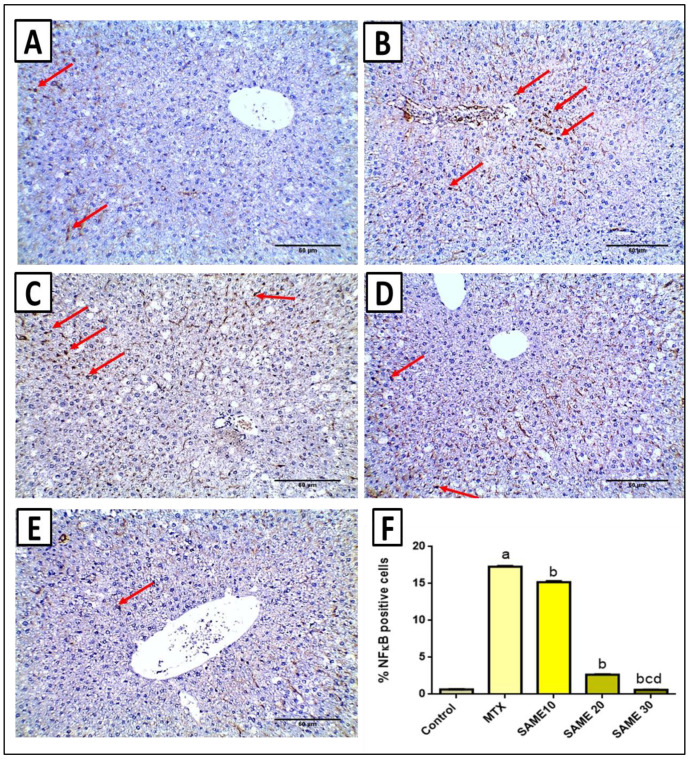
Effects of the SAME pretreatment on immunohistochemical staining of NF-κB. (**A**) A section in the liver of the normal control group showed negative immunostaining in hepatocytes (nuclear positivity in less than 1% of hepatocytes) (red arrows) [×200]. (**B**) A section in the liver of the MTX group showed positive nuclear staining in more than 10% of hepatocytes score 2 (red arrows) [×200]. (**C**) A section in the liver of the SAME 10 pretreated group showed positive nuclear staining in more than 10% of hepatocytes score 2 (red arrows) [×200]. (**D**) A section in the liver of the SAME 20 pretreated group showed positive nuclear staining in 1–10% of hepatocytes score 1 (red arrows) [×200]. (**E**) A section in the liver of the SAME 30 pretreated group showed negative immunostaining in hepatocytes (nuclear positivity in less than 1% of hepatocytes) (red arrows) [×200]. (**F**) Percentage of positive cells/1000 cells per 10 high power fields. Data expressed as mean ± SD. Significant difference vs. ^a^ respective control, ^b^ respective MTX group, ^c^ respective SAME 10 group, ^d^ respective SAME 20 group each at *p* < 0.05.

NF-κB plays an essential role in the pathophysiology of MTX-induced hepatotoxicity, so it was assessed and quantified by immunostaining. Immunostaining of the nucleus of hepatocytes is a unique way to determine the positivity of NF-κB expression. Staining in blood sinusoids was considered negative. A section in the liver of the control saline group presented negative immunostaining in hepatocytes (nuclear positivity in less than 1% of hepatocytes) (Figure 9A). However, a section in the liver of the MTX group showed positive nuclear staining in more than 10% of hepatocytes score 2 (Figure 9B). Furthermore, the section in the liver of the SAME 10 group showed positive nuclear staining in more than 10% of hepatocytes score 2 (Figure 9C). A section in the liver of SAME 20 group showed positive nuclear staining in 1–10% of hepatocytes score 1 (Figure 9D). In addition, the section in the liver of the SAME 30 group showed negative immunostaining in hepatocytes (nuclear positivity in less than 1% of hepatocytes) (Figure 9E). Results of immune-staining quantification revealed that the control group showed very weak NF-κB immunostaining. MTX significantly elevated NF-κB immunostaining by 26.21-fold compared to the control group and treatment with SAME induced a marked suppression of NF-κB staining by 12.25, 84.59, and 96.53%, respectively, relative to the MTX group (Figure 9F, *p* < 0.05).

### 2.7. Histopathological Studies

**Figure 10:**
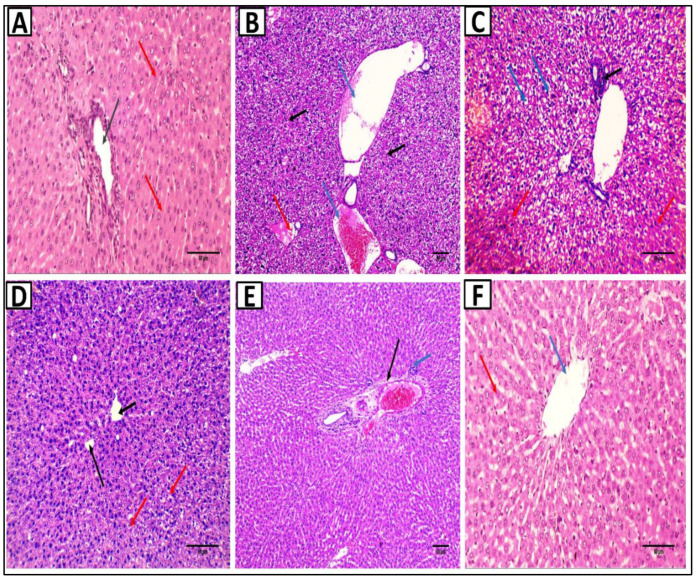
Histopathological examination of the liver sections stained with hematoxylin and eosin (H&E). (**A**) A section in the liver of the control group showed portal tract (portal venule, hepatic arteriole, and bile ductule) (black arrow) surrounded by cords of hepatocytes (red arrows) [H&E × 200]. (**B**): Section in the liver of MTX group showed congested central vein (red arrow), dilated portal venule, and hepatic arteriole (blue arrows) surrounded by cords of hepatocytes showing focal necrosis (black arrows) [H&E × 100]. (**C**): Section in the liver of MTX group showed portal tract showing chronic inflammatory cellular infiltrate (black arrow) surrounded by cords of hepatocytes showing hydropic degeneration (blue arrows) and focal necrosis (red arrows) [H&E × 200]. (**D**): Section in the liver of SAME 10 group showed average-sized central veins (black arrows) surrounded by cords of average-sized hepatocytes, some of which showed hydropic degeneration (red arrows) [H&E × 200]. (**E**): Section in the liver of the SAME 20 group showed mild dilated portal venule (black arrow) surrounded by a few chronic inflammatory (blue arrow) surrounded by cords of average-sized hepatocytes with no necrosis or degeneration [H&E × 100]. (**F**): Section in the liver of the SAME 30 group showed an average-sized central vein (blue arrow) surrounded by cords of hepatocytes (red arrow) with no necrosis, degeneration, or inflammation [H&E × 200].

Liver slices from the normal control group were examined histopathologically and illustrated portal tract (portal venule, hepatic arteriole, and bile ductule) surrounded by cords of hepatocytes (Figure 10A). In contrast, liver sections of the MTX group showed a congested central vein, dilated portal venule, and hepatic arteriole surrounded by cords of hepatocytes showing focal necrosis (Figure 10B). In addition, the portal tract had chronic inflammatory cellular infiltrates surrounded by cords of hepatocytes with hydropic degeneration and focal necrosis (Figure 10C). The sections in the liver of the SAME 10 group showed average-sized central veins surrounded by cords of average-sized hepatocytes, some of which showed hydropic degeneration (Figure 10D). Furthermore, the liver sections of the SAME 20 group showed mild dilated portal venule surrounded by few chronic inflammatory cells and surrounded by cords of average-sized hepatocytes with no necrosis or degeneration (Figure 10E). Liver sections of the SAME 30 group showed an average-sized central vein surrounded by cords of hepatocytes with no necrosis, degeneration, or inflammation **(**Figure 10F and Table 5).

## 3. Discussion

Acute liver injury (ALI) is a potentially fatal condition marked by significant inflammation, contributing to a greater death rate in hospitals [17]. MTX-hepatotoxicity is one of the most common adverse effects that limit its clinical usage. Our study aims to assess the possible hepato-protective effects of SAME against MTX-associated hepatotoxicity and to explore the underlying mechanisms of such protective effects. As far as we know, this is the earliest research investigating these effects.

The current study found that mice given MTX had a considerable increase in ALT, AST, and LDH, indicating a severe liver injury. A rise in their concentration in the blood implies a leak in the cell membrane, which is linked to hepatocyte mortality [15].

The results of the histological examination, which revealed significant liver damage in the MTX group, corroborated these biochemical abnormalities. Treatment with SAME considerably reduced these biochemical and histological alterations implying that SAME might effectively reverse MTX-induced liver cell damage.

Inflammation has a role in tissue homeostasis and the pathophysiology of various diseases involving acute or chronic liver damage [18]. The NLPR3 inflammasome is a major inflammation signaling system triggered by injury or pathogen-associated molecular patterns. The cytokine precursors pro-IL-1 and pro-IL-18 are converted into mature and physiologically active IL-1 and IL-18, respectively, by NLPR3 triggering proteolytic cleavage of dormant pro-caspase-1 into active caspase-1 [17], which further exacerbates the inflammatory process. Accordingly, it is reported that NLPR3 inflammasome activation results in severe liver inflammation and fibrosis and can serve as a therapeutic target [19,20,21].

However, it is unclear whether SAME affects MTX-induced ALI and whether it is linked to the Nrf2 antioxidant pathway or NLPR3 inflammasome activation. This was the first study to look into SAME’s protective impact and mechanism of action in mice with MTX-induced ALI.

Based on the data obtained from LC/MS, SAME contained several major compounds such as chlorogenic acid, trans ferulic acid, baicalin, apigenin, and vitexin, which have been clearly reported previously to inhibit the NLPR3 inflammasome pathway.

In the current study, the NLPR3 inflammasome pathway is strongly activated, manifested by striking elevations in NLPR3 expression and caspase-1/IL-1β gene expression. SAME induced a marked down-regulation of the NLPR3/caspase-1/IL-1β signaling axis in a dose-dependent way; these inhibitory effects of this extract are attributed to its major components, including chlorogenic acid, *Trans* ferulic acid, baicalin, apigenin, and vitexin, which are natural NLPR3 inflammasome inhibitors as reported previously [16,22,23,24,25,26,27,28]. Inhibition of the inflammasome pathway by SAME provides brilliant therapeutic potential for protection against MTX-induced hepatotoxicity.

Pro-inflammatory cytokines such as IL-1 and IL-18, according to new findings, play a critical role in hepatic damage [17,29]. Our study shows that the hepatic pro-inflammatory cytokines, IL-1β and IL-18, which are downstream molecules of the NLPR3 axis, are increased significantly. The SAME pretreatment induced a dose-dependent inhibition in inflammatory cytokines levels. These findings suggested that SAME’s anti-inflammatory action on the liver injury was due to NLPR3 signaling pathway suppression. Thus, our findings confirmed that the NLPR3 axis is vital in ALI, and SAME protects against MTX-induced ALI via inhibiting the generation of pro-inflammatory cytokines mediated by NLRP3.

Several investigations have explored the significant role that oxidative stress plays in the pathophysiology of chronic and ALI caused by various etiologies [30]. The reactive oxygen species (ROS) can destroy polyunsaturated fatty acids and cause lipid peroxidation and production of MDA [31], which is considered an indicator for the total level of lipid peroxidation. They also deplete the tissue antioxidant enzyme capacity, making the tissue more vulnerable to the toxic free radicals’ effects. In the present study, SAME significantly reduced MDA NO and restored SOD levels in the liver tissue. Accordingly, SAME could inhibit MTX-induced oxidative stress in the liver. As reported previously, these effects are ascribed to the major components, including chlorogenic acid, trans ferulic acid, baicalin, apigenin, and vitexin [17,23,24,25,26,27,28].

Nrf2 is believed to be a key endogenous oxidative stress regulator [27,32]. By promoting its target genes, Nrf2 has a variety of consequences in the liver, including inflammation, fibrosis, cancer, and regeneration [33]. When fed a high-fat diet, Nrf2-null mice develop hepatic steatosis and inflammation higher than wild-type mice [34]. As a result, Nrf2 is regarded as a pharmacological target for preventing and treating a variety of hepatic illnesses [27]. Our research revealed that SAME significantly up-regulated Nrf2 gene expression and enhanced the antioxidant enzymes as SOD. Thus, SAME could prevent MTX-induced hepatotoxicity via attenuation of oxidative stress.

Oxidative stress has been demonstrated to play a key influence in activating the NLRP3 inflammasome in earlier studies [35,36]. Hence, The NLPR3 inflammasome can be turned off by inhibiting oxidative stress. The current research displayed that SAME considerably induced Nrf2 expression and inhibited hepatic oxidative damage caused by MTX. These findings suggested that SAME’s inhibitory action on NLRP3 inflammasome activation could be mediated by regulating the Nrf2 antioxidant pathway.

SAME’s various components have been investigated for links to a positive effect, including chlorogenic acid, baicalin, ferulic acid, vitexin, and apigenin [16,17,22,23,24,25,26,27,28]. The most crucial component is chlorogenic acid, reported previously as an inhibitor to Nrf2 [27,37,38,39,40]. Several recent studies have offered evidence that chlorogenic acid, baicalin, ferulic acid, and apigenin can manage Nrf2/ARE signaling pathways, thus preventing carcinogenesis [26,27,28].

The present study revealed that prior SAME treatment might reduce the severity of MTX-induced ALI by inhibiting oxidative stress and NLPR3 inflammasome activation. As a result, SAME has the potential to be a powerful hepatoprotective substance in the prevention of oxidative stress-associated liver damage.

The nuclear transcription factor NF-κB is involved in the pathophysiology of MTX-induced hepatotoxicity. As a result of oxidative stress, NF-κB is released and translocated into the nucleus. Accordingly, it attaches to DNA and increases the expression of inflammatory genes such as cytokine and chemokine [30]. In the current study, SAME treatment induced a marked activation of the NF-κB pathway upon MTX administration, which is in line with a previously reported study [41]. Pretreatment with SAME showed a remarkable inhibitory effect on the NF-κB activation pathway. This effect is attributed to SAME’s significant components, including baicalin and apigenin, which are NF-κB inhibitors [42,43]. For the first time, these findings showed that SAME’s hepatoprotective properties are likely to be linked to its ability to control NF-κB.

The current research finding investigated that MTX induced significant hepatocytes apoptosis manifested by marked up-regulation of caspase-3 levels in the hepatic tissue. This effect was substantially mitigated by prior treatment with SAME. These findings are comparable to a previous study investigating SAME’s capacity to decrease apoptosis [38].

Resistance of the pathogenic bacteria to antibiotics was first detected in the 1940s. Since then, this problem has continued to increase and evolve [44]. Currently, antibiotic resistance is highly spreading among *A. baumannii*, and this is a therapeutic challenge that should be addressed and considered [12]. Herein, we found that 77.78% of *A. baumannii* isolates were multidrug-resistant. Many studies have reported on the high rate of multidrug-resistant *A. baumannii* in hospital settings, especially among patients suffering from liver diseases [45,46,47]. This high rate of multidrug resistance needs prompt action to decrease the morbidity and mortality of the infections caused by these bacteria. Plants are considered a huge source of many therapeutic compounds which need more exploration and investigation. Thus, we investigated the antibacterial activity of SAME against *A. baumannii* clinical isolates, and we studied its antibacterial mode of action.

The bacterial cell membrane is a crucial selectively permeable barrier that can organize the exchange between the internal and external environments. The loss of this property usually leads to cell death [48]. Here, the membrane integrity was studied via monitoring the release of DNA and RNA from the cell over time by measuring the absorbance at 260 nm. A considerable reduction in membrane integrity was noticed in 51.85% of the isolates treated with SAME. In addition, the protein content in the culture media was quantified by the Biuret method. We observed that SAME has resulted in an essential increase in protein in 48.15% of *A. baumannii* isolates.

The outer membrane is characteristic for Gram-negative bacteria only, and it is regarded as an extra barrier that inhibits the penetration of various toxic compounds, such as antibiotics [49]. Herein, the permeability of the outer and inner membranes was significantly increased in 55.56% and 59.26% of *A. baumannii* isolates, respectively.

Examination of the bacterial cells, before and after treatment with SAME, using SEM was carried out in the current study to understand the influence of SAME on the morphology and ultrastructure of *A. baumannii* isolates. Interestingly, we observed that SAME treatment has resulted in disruption of the cell wall and lysis of some bacterial cells, a finding that is consistent with the effects of SAME on the membrane integrity, outer, and inner membrane permeability. Many studies have reported the harmful impact of different plant extracts on the morphology of bacterial cells [34,50,51].

## 4. Materials and Methods

### 4.1. Animals

The animal house at Cairo University provided forty male albino mice (weighing 22–26 g) (Cairo, Egypt). Mice were housed in rat cages with a regular pellet meal and filtered water and were kept at a constant temperature (25 °C) and light (12h light/dark cycle). All the mice had been acclimated before use in experiments for seven days. The experimental protocols and procedures were conducted in compliance with the recommendations for laboratory animal care and service. They were given the approval number PO-2021-00107 (25 August 2021) by the Research Ethical Committee (Faculty of Pharmacy, Tanta University, Tanta, Egypt).

### 4.2. Plant Materials and Extract Preparation

Taxonomic nomenclature of *S. auriculata* was according to [52,53]. Specimens of *S. auriculata* Aubl. were collected and identified at the Herbarium of the Botanic Garden (Heneidy Collection), Alexandria University, Alexandria, Egypt, where they were deposited. The plant powder (260 g) was extracted using 100% methanol, adopting a cold maceration method (3 × 2 L). The extract was concentrated using a rotary vacuum evaporator to obtain a residue (30.2 g).

### 4.3. Drugs and Chemicals

MTX vial 50 mg/2 mL (Mylan Pharmaceuticals, Canonsburg, PA, USA) was used in this study. The remaining chemicals and solvents were bought from Sigma-Aldrich and were of high-quality grade (St. Louis, MO, USA).

### 4.4. Bacterial Isolates

A total of 27 *A. baumannii* clinical isolates were collected from patients suffering from liver diseases in intensive care units of Tanta University Hospitals. They were identified using microscopical examination and standard biochemical tests [54].

### 4.5. LC-ESI-MS/MS for Metabolite Profiling

SAME was reconstituted in distilled water, methanol, and acetonitrile 50:25:25. It was analyzed using an ExionLC^TM^ AD UPLC and a TripleTOF 5600+ Tandem Mass Spectrometer (AB SCIEX). They adopted the criteria described previously [55,56]. To identify compounds, PeakView 2.2 with the MasterView 1.1 software (AB SCIEX) was used to compare *m*/*z* values obtained by MS and MS^2^. The XIC Manager in PeakView^TM^ software calculated the peak area values. Extracted ion chromatograms (XICs) for each targeted analyte were automatically created and compared to a user-defined threshold.

### 4.6. In Vitro Antibacterial Activity

#### 4.6.1. Antibiotic Susceptibility Testing

According to the Clinical and Laboratory Standards Institute’s guidelines, the disc diffusion method was used [57]. In brief, overnight bacterial cultures were spread on the surface of Muller-Hinton agar plates (HiMedia, Mumbai, India), then the antibiotic discs were put on the surfaces of the plates. The utilized antibiotics were: cephalothin (30 μg), cefuroxime (30 μg), ceftazidime (30 µg), cotrimoxazole (23.75/1.25 μg), gentamicin (10 µg), amikacin (30 µg), tobramycin (10 µg), azithromycin (15 µg), tetracycline (30 μg), chloramphenicol (30 μg), ciprofloxacin (5 μg), levofloxacin (5 µg), and imipenem (10 μg).

#### 4.6.2. Screening of the Antibacterial Activity of SAME

This stage was conducted using the well diffusion method [56]. Briefly, 100 µL of overnight bacterial suspensions were spread on the surface of Muller-Hilton agar plates. Then, using a cork-borer, three wells were punched off the agar. The first well was filled with 100 μL of SAME (1024 µg/mL), the second well was filled with DMSO (negative control), and the third well was filled with ciprofloxacin (positive control) [58]. Finally, the plates were overnight incubated at 37 °C.

#### 4.6.3. MIC

The values of MICs of SAME were detected by the broth microdilution method in 96 well microtitration plates as previously reported [59]. Each plate had a positive control well (bacterial suspension without SAME) and a negative control well (broth only). The values of MICs for each bacterial isolate were determined as the lowest concentration of SAME that inhibited the growth (indicated by the absence of turbidity). The subsequent tests were conducted before and after treating *A. baumannii* isolates with 0.5 MIC values.

#### 4.6.4. Mode of Action of the Antibacterial Activity

- Loss of cellular content

After centrifuging overnight cultures of *A. baumannii* isolates, the obtained pellets were washed twice, resuspended in saline solution, and incubated (before and after treatment with SAME) at 37 °C for 24 h in a shaker incubator. A sample (1 mL) was taken every one hour, centrifuged, and the supernatant absorbance was measured at 260 nm [60].

b. Effect on membrane permeability

We examined outer and inner membrane permeability in the investigated isolates as described [61]. NPN was used to assess the outer membrane permeability. Overnight bacterial cultures (before and after treatment with SAME) were thoroughly blended with NPN solution (20 μmol) and incubated at 37 °C. The NPN fluorescence was determined over time at an emission wavelength of 340 nm and excitation wavelength of 420 nm using a florescent spectrofluorometer (Shimadzu, Kyoto, Japan).

The inner membrane permeability was evaluated in *A. baumannii* isolates using ONPG, which is degraded by a β-galactosidase enzyme. This was done by observing the release of the β-galactosidase enzyme from the bacterial cells to the outside. Briefly, after centrifuging overnight bacterial cultures (before and after treatment with SAME) in Mueller–Hinton broth with 2% lactose, the pellets were washed and resuspended in sodium chloride solution (0.5%). Finally, ONPG (34 mmol) was added to the bacterial suspensions, and the optical density (OD) was recorded at 420 nm.

c. Estimation of the protein content

Protein content was quantified by the Biuret method as previously described [62]. In brief, overnight cultures of *A. baumannii* isolates (before and after treatment with SAME at 0.5 MIC values) were incubated for 24 h at 37 °C in a shaker incubator. Samples were taken at time intervals of 2 h, and they were centrifuged for 10 min at 10,000 rpm. Then, 0.5 mL was taken from each sample supernatant, and 5 mL of the biuret reagent was added and left for 20 min. The absorbance of the test samples and the standard (serum bovine albumin) was then measured at an OD of 550 nm (OD_550_). The protein concentrations were calculated according to the equation:

(1) $$\mathrm{Protein}\;\mathrm{concentration}\;\left(\mathrm{mg}/100\;\mathrm{ml}\right)=\frac{A\;\mathrm{sample}}{A\;\mathrm{standard}}\times 5$$

d. Scanning electron microscopy (SEM)

SEM was utilized to explore the impact of SAME on the ultrastructure and the morphological features of *A. baumannii*, as previously described. After fixing the bacterial cells with glutaraldehyde (4%) and osmic acid solution (1%), the samples were sequentially dehydrated in 30–95% cold ethanol. Then, they were dehydrated in absolute ethanol twice for 20 min, left to dry under CO_2_, and examined using SEM (Joel-1200, ECII, Akashi Seisakusho, Japan) [63].

### 4.7. In Vivo Hepatoprotective Activity

#### 4.7.1. Experimental Design

Five groups of mice (8 animals each) were assigned at random as the following:

Group I: a control group in which mice were given regular saline until the experiment was completed. Group II: MTX group was given a single intraperitoneal (I.P) injection of MTX (20 mg/kg) on the 6th day. [14]. Groups 3, 4, and 5: mice were given SAME at three dose levels (10, 20, and 30 mg/kg orally) once daily for five days and a single injection of MTX (20 mg/kg, I.P) on the 6th day. Due to the lack of in vivo studies on SAME, the highest protective dose against MTX-induced hepatotoxicity was determined by comparing three doses. These doses were selected depending on Sirlaxmi et al. previous study [64].

#### 4.7.2. Sample Collection

On day 7, the animals were anesthetized by diethyl ether inhalation, and a heart puncture was used to collect blood samples. After that, the blood was centrifuged at 3000 rpm for 10 min. The serum was appropriately separated and stored at −20 °C until required. Under light ether anesthesia, mice were then sacrificed by cervical dislocation. Liver tissues were removed, snap-frozen, washed with ice-cold phosphate-buffered saline, and kept at −80 °C for further assessments. For histological and immunohistochemical investigation, a liver section was also excised and stored in a 10% neutral buffered formalin solution.

#### 4.7.3. Serum Indices of Hepatotoxicity

An alanine aminotransferase colorimetric activity assay kit (Cayman, USA, 700260), aspartate aminotransferase colorimetric activity assay kit (Cayman, Michigan, USA, 701640), and LDH Assay Kit #37291(cell signaling) were used to estimate AST, ALT, and LDH according to manufacturer protocol using double beam spectrophotometer (Shimadzu, Kyoto Japan).

#### 4.7.4. Assessment of Lipid Peroxidation

Malondialdehyde levels in the liver tissue homogenate were tested using commercially available kits to determine the level of lipid peroxidation (Biodiagnostic, Giza, Egypt).

#### 4.7.5. Measurement of Liver Nitric Oxide Levels

As previously mentioned [29], the NO level in liver tissue homogenate was evaluated by detecting its stable metabolites, nitrite and nitrate. Griess reagent can be used to determine these anions colorimetrically. The absorbance at 540 nm was measured using a Shimadzu spectrophotometer.

#### 4.7.6. Assessment of SOD Activity

Using a commercially available kit (Biodiagnostic, Giza, Egypt), the activity of the SOD enzyme in the liver homogenate was determined following the manufacturer’s instructions.

#### 4.7.7. Quantitative Real-Time Polymerase Chain Reaction (qRT-PCR)

The reaction was performed to examine the expression of the Nrf2, IL-1β, IL-18, caspase-1, and caspase-3. The TRIzol reagent (Life Technologies, Inc., Carlsbad, CA, USA) purified total RNA from liver samples following the manufacturer’s procedure. NanoDrop ND-1000 estimated RNA yield and purity (Nanodrop technologies, Wilmington, DE, USA). RNA samples with an absorbance ratio OD 260/280 between 1.9–2.2 and OD 260/230 greater than 2.0 were used for further analysis. Furthermore, agarose gel electrophoresis was applied for assaying RNA integrity. 

Total RNA (1 µg) was reverse-transcribed into single-stranded complementary DNA (cDNA) using the QuantiTects Reverse Transcription Kit (Qiagen, Germantown, MD, USA) and a random primer hexamer in a two-step RT-PCR experiment. All reverse-transcription experiments include negative control to test for contaminating genomic DNA. Negative control contains all components except for the template RNA.

Maxima SYBR Green/Fluorescein qPCR Master Mix (Thermo Scientific, Waltham, MA, USA) was used to amplify cDNA amplicons using primers (Supplementary Table S2) produced following the manufacturer’s procedure. 

Thermal cycling conditions were set at 95 °C for 10 min, then 10 s at 95 °C, 15 s at 60 °C, and 15 s at 72 °C for 45 cycles. The melting curve analysis was carried out between 72 and 95 degrees Celsius, with a temperature increase of 1.0 degrees Celsius per second. On each sample, RT-PCR was done in duplicate, and the mean values of the duplicates were used for further analysis. Finally, the 2^−ΔΔCT^ method was used to calculate relative mRNA expression, then standardized to GAPDH [65].

#### 4.7.8. Western Blot Analysis for NLPR3 Inflammasome

Total soluble proteins from the samples were fragmented on 10% sodium dodecyl sulfate–polyacrylamide gels, then moved to a HybondTM nylon membrane (Merck, Kenilworth, NJ, USA) using a TE62 Standard Transfer Tank with Cooling Chamber (Hoefer Inc., San Diego, CA, USA) and incubated for one hour at room temperature. Then, using β-actin as a housekeeping protein, 5% skim milk (BD, Franklin Lakes, NJ, USA) was used as a blocking solution for 60 min. The membrane has incubated overnight at 4 °C in an antibody solution containing an Anti-NLRP3 antibody [EPR23094-1] (Abcam, MA, USA). For triplicate washing, phosphate-buffered saline solution with a low concentration of detergent solution, such as 0.05 percent to 0.1 percent tween 20 (PBST), was then incubated with HRP-conjugated secondary antibody (antibody concentration 0.1–0.5 µg/mL) for one hour. To achieve the required signal strength and low background, the antibody concentration was varied from 0.05 to 2.0 µg/mL. Finally, an ECL western blotting substrate was used to detect the signal (Protein Simple, FluorChem E).

#### 4.7.9. Histopathological Examination of Liver Sections

The liver was sectioned at a thickness of 4 µm in paraffin blocks, stained with H&E, and inspected under a light microscope. The semi-quantitative ranking was utilized to evaluate the overall assessed value of the histopathological lesion following the previously stated methodology [66]. The degree and scope of the change are graded on a scale of 0 to 3, as previously indicated [66].

#### 4.7.10. Immunohistochemical Staining of NF-κB 

A 10% neutral formalin solution was used to fix the liver samples. They were bisected, paraffin treated, and then sectioned into 5 µm-thick layers after 24 h. The 5 µm-thick pieces were dewaxed and rehydrated with D.W. after being mounted on glass slides and stained with NF-κB (ABclonal Technology, Woburn, MA, USA). Only distinct nuclear immune-staining in hepatocytes termed activated NF-κB was used to determine NF-ĸB expression positivity. Staining in blood sinusoids was considered negative. Nuclear localization of NF-κB was classified as negative [0 <1%] or positive and scored as the overall proportion of cells [1–10% (score 1), and >10% (score 2)] with positive nuclear staining in the investigated field at 200 magnification [67]. Immune-stained slides were image analyzed using Image J software. The staining scores were calculated by the percentage of positive cells within 1000 cells being counted on each slide in the area of maximum staining per 10 high power fields after background substraction.

### 4.8. Statistical Analysis

The data was provided as a mean ±standard deviation. Regression analysis was performed on all standard curves, producing correlation coefficients. A one-way analysis of variance (ANOVA) was utilized to compare different groups, followed by a Tukey–Kramer posthoc test. The *p* < 0.05 significance level was set. The statistical analysis was conducted using Prism version 9. (GraphPad Software, Inc., San Diego, CA, USA).

## 5. Conclusions

Collectively, our results displayed for the first time that pretreatment with *S. auriculata* extract provides a promising protective effect against MTX-induced hepatotoxicity. This protective effect could be due to reducing oxidative stress and NLPR3 inflammasome axis activation. It also exerted a remarkable anti-inflammatory and anti-apoptotic activity. Hence, SAME has the potential to be a powerful hepatoprotective therapy for the amelioration of oxidative stress-mediated liver damage. In addition, SAME exhibited antibacterial activity against *A. baumannii* clinical isolates. The mechanism of its antibacterial action involved its ability to decrease the membrane integrity, increase the outer and inner membrane permeability, and produce morphological changes in the studied bacterial isolates. Therefore, SAME could be a promising antibacterial compound. However, more investigations are required to ensure the therapeutic efficacy and safety of SAME to be used as an antimicrobial and hepatoprotective agent.

## Figures and Tables

**Table 1:** Phytochemical profiling of SAME by LC-ESI-MS/MS in negative and positive mode.

No.	Rt (min)	Precursor *m*/*z*	Error ppm	Metabolite Name	Formula	Adduct	MS/MS Spectrum	Class
1	1.36	179.034	0.5	Caffeic acid	C_9_H_8_O_4_	[M-H]^−^	107.050 [C_7_H_5_O+2H]^−^, 117.034 [C_8_H_6_O]−H^−^, 135.045 [C_8_H_7_O_2_]^−^	Hydroxy cinnamic acids
2	1.42	181.049	0.4	Caffeic acid	C_9_H_8_O_4_	[M+H]^+^	53.002 [C_3_H_2_O−H]^+^, 122.036 [C_7_H_6_O_2_]^+^, 137.059 [C_8_H_7_O_2_+H]+H^+^, 163.038 [C_9_H_7_O_3_]^+^	Hydroxy cinnamic acids
3	2.14	169.097	0.1	Pyridoxamine	C_8_H_12_N_2_O_2_	[M+H]^+^	70.065 [C_4_H_6_N+H]+H^+^, 86.060 [C_4_H_7_NO]+H^+^, 124.063 [C_6_H_6_N_2_O+H]+H^+^	Pyridoxamine 5’-phosphates
4	3.76	341.086	5.3	Esculin	C_15_H_16_O_9_	[M+H]^+^	108.02 [C_6_H_3_O_2_]+H^+^, 123.007 [C_6_H_3_O_3_]^+^, 137.023 [C_7_H_3_O_3_+H]+H^+^, 151.060 [C_5_H_10_O_5_]+H^+^, 179.055 [C_6_H_11_O_6_]^+^	Coumarin glycosides
5	4.22	147.045	0.1	Trans- Cinnamate	C_9_H_8_O_2_	[M-H]^−^	77.039 [C_6_H_5_]^−^, 103.055 [C_8_H_7_]^−^, 119.050 [C_8_H_6_O+H]^−^	Cinnamic acids
6	4.747	195.087	−0.8	Caffeine	C_8_H_10_N_4_O_2_	[M+H]^+^	110.071 [C_5_H_7_N_3_]+H^+^, 138.066 [C_6_H_7_N_3_O]+H^+^, 151.037 [C_6_H_4_N_3_O_2_]+H^+^	Xanthines
7	5.01	154.049	−0.8	3-hydroxy anthranilic acid	C_7_H_7_NO_3_	[M+H]^+^	94.02 [C_6_H_4_O+H]+H^+^, 112.03 [C_5_H_5_NO_2_]+H^+^, 122.02 [C_7_H_4_O_2_+H]+H^+^, 140.03 [C_6_H_6_NO_3_]^+^	Hydroxy benzoic acid derivatives
8	5.07	177.019	−0.6	6,7-dihydroxy coumarin	C_9_H_6_O_4_	[M-H]^−^	77.039 [C_6_H_4_+H]^−^, 105.034 [C_7_H_4_O+H]^−^, 133.029 [C_8_H_6_O_2_]−H^−^, 149.024 [C_8_H_6_O_3_]−H^−^	6,7-dihydroxy coumarins
9	5.198	355.102	−0.9	Chlorogenic acid (3-caffeoylquinic acid)	C_16_H_18_O_9_	[M+H]^+^	89.02 [C_3_H_4_O_3_]+H^+^, 117.018 [C_4_H_6_O_4_−H]^+^, 135.044 [C_8_H_7_O_2_]^+^, 145.049 [C_6_H_10_O_4_−H]^+^, 163.038 [C_9_H_7_O_3_]^+^, 337.091 [C_16_H_17_O_8_]^+^	Quinic acids and derivatives
10	5.296	179.033	0.6	6,7-dihydroxycoumarin (esculetin)	C_9_H_6_O_4_	[M+H]^+^	51.02 [C_4_H_3_]^+^, 77.03 [C_6_H_5_]^+^, 105.03 [C_7_H_5_O]^+^, 123.04 [C_7_H_6_O_2_]+H^+^, 133.02 [C_8_H_5_O_2_]^+^, 135.02 [C_8_H_5_O_2_+H]+H^+^, 151.0 [C_8_H_6_O_3_]+H^+^	6,7-dihydroxy coumarins
11	5.3	179.033	0.6	Daphnetin	C_9_H_6_O_4_	[M+H]^+^	77.038 [C_6_H_4_]+H^+^, 104.997 [C_6_H_3_O_2_−2H]^+^, 123.007 [C_6_H_4_O_3_−H]^+^, 133.028 [C_8_H_6_O_2_−H]^+^, 135.044 [C_8_H_6_O_2_]+H^+^, 151.038 [C_8_H_6_O_3_]+H^+^	7,8-dihydroxy coumarins
12	5.352	353.087	−0.4	Chlorogenic acid	C_16_H_18_O_9_	[M-H]^−^	111.045 [C_6_H_5_O_2_+2H]^−^, 135.045 [C_8_H_7_O_2_]^−^, 161.024 [C_9_H_7_O_3_−H]−H^−^, 173.045 [C_7_H_11_O_5_−H]−H^−^, 179.035 [C_9_H_7_O_4_]^−^, 191.0561 [C_7_H_11_O_6_]^−^	Quinic acids and derivatives
13	6.172	255.065	−0.8	Daidzein	C_15_H_10_O_4_	[M+H]^+^	91.017 [C_6_H_4_O−H]^+^, 131.049 [C_9_H_5_O+H]+H^+^, 181.028 [C_12_H_6_O_2_−H]^+^, 182.036 [C_12_H_6_O_2_]^+^, 199.038 [C_12_H_7_O_3_]^+^, 219.044 [C_15_H_8_O_2_−H]^+^, 237.054 [C_15_H_9_O_3_]^+^	Isoflavones
14	7.16	193.049	−0.1	Scopoletin	C_10_H_8_O_4_	[M+H]^+^	77.038 [C_6_H_4_]+H^+^, 122.036 [C_7_H_6_O_2_]^+^, 133.028 [C_8_H_5_O_2_]^+^, 137.059 [C_8_H_7_O_2_+H]+H^+^, 150.031 [C_8_H_5_O_3_]+H^+^, 178.026 [C_9_H_5_O_4_]+H^+^	7-hydroxy coumarins
15	7.65	433.112	4.5	Apigenin 8-C-glucoside (vitexin)	C_21_H_20_O_10_	[M+H]^+^	239.0346 [C_11_H_11_O_6_]^+^, 267.0287 [C_12_H_11_O_7_]^+^, 295.0241 [C_13_H_11_O_8_]^+^, 313.0346 [C_13_H_14_O_9_−H]^+^	Flavonoid 8-C-glycosides
16	8.32	447.092	1.4	Baicalein-7-*O*-glucuronide	C_21_H_18_O_11_	[M+H]^+^	117.069 [C_9_H_6_+2H]+H^+^, 239.033 [C_14_H_9_O_4_−2H]^+^, 267.049 [C_12_H_9_O_7_+H]+H^+^, 295.044 [C_13_H_11_O_8_]^+^, 313.055 [C_13_H_11_O_9_+H]+H^+^	Flavonoid-7-*O*-glucuronides
17	8.41	463.088	0.7	6-hydroxy kaempferol 3-glucoside	C_21_H_20_O_12_	[M-H]^−^	301.035 [C_15_H_9_O_7_]^−^, 315.051 [C_16_H_10_O_7_+H]^−^, 343.045 [C_17_H_12_O_8_]−H^−^, 403.067 [C_19_H_16_O_10_]−H^−^	Flavonoid-3-*O*-glycosides
18	8.68	195.065	−0.2	Trans-ferulic acid	C_10_H_10_O_4_	[M+H]^+^	63.02 [C_5_H_4_−H]^+^, 79.05 [C_5_H_3_O]^+^, 117.03 [C_8_H_6_O−H]^+^, 135.04 [C_8_H_6_O_2_]+H^+^, 145.02 [C_9_H_6_O_2_−H]^+^, 163.03 [C_9_H_7_O_3_]^+^	Hydroxy cinnamic acids
19	8.76	449.108	0.3	Cyanidin 4″-glucoside	C_21_H_21_O_11_	[M] ^+^	147.065 [C_6_H_10_O_4_]+H^+^, 252.062 [C_12_H_14_O_6_−2H]^+^, 273.096 [C_12_H_15_O_7_+H]+H^+^	Anthocyanidin -3-*O*-glycosides
20	9.742	287.055	0.9	Luteolin	C_15_H_10_O_6_	[M+H]^+^	135.044 [C_8_H_6_O_2_]+H^+^, 153.018 [C_7_H_4_O_4_]+H^+^, 215.070 [C_13_H_8_O_3_+2H]+H^+^, 255.065 [C_15_H_8_O_4_+2H]+H^+^, 269.044 [C_15_H_9_O_5_]^+^	Flavones
21	10.52	269.045	0.6	Apigenin	C_15_H_10_O_5_	[M-H]^−^	117.034 [C_8_H_6_O]−H^−^, 149.024 [C_8_H_5_O_3_]^−^, 183.045 [C_12_H_6_O_2_+H]^−^, 225.055 [C_14_H_9_O_3_]^−^, 227.035 [C_13_H_9_O_4_−H]-H^−^, 254.058 [C_15_H_9_O_4_+H]^−^	Flavones
22	10.76	211.132	−0.3	(+-)-Jasmonic acid	C_12_H_18_O_3_	[M+H]^+^	81.1 [C_6_H_10_−H]^+^, 123.1 [C_8_H_10_O]+H^+^, 147.03 [C_11_H_17_−2H]^+^, 157.1 [C_8_H_11_O_3_+H]+H^+^, 165.1 [C_11_H_17_O]^+^	Jasmonic acids
23	10.88	271.060	0.9	Apigenin	C_15_H_10_O_5_	[M+H]^+^	119.08 [C_8_H_6_O]+H^+^, 153.02 [C_7_H_4_O_4_]+H^+^, 253.14 [C_15_H_9_O_4_]^+^	Flavones
24	10.89	271.060	0.9	Genistein	C_15_H_10_O_5_	[M+H]^+^	119.049 [C_8_H_6_O]+H^+^, 153.018 [C_7_H_4_O_4_]+H^+^, 243.065 [C_12_H_10_O_4_]^+^, 253.049 [C_15_H_9_O_4_]^+^	Isoflavones

**Table 2:** Antimicrobial resistance profile of *A. baumannii* isolates.

Isolate Number	Resistance Profile *	Isolate Number	Resistance Profile *
A1	CEF- SXT-GEN-AMK	A15	CEF-SXT-CIP
A2	CEF-CXM-TOB-CIP	A16	CXM-TET-CHL-CIP
A3	CXM-GEN-CHL	A17	CEF-SXT-GEN
A4	AZM-TET	A18	SXT-GEN-AMK-TOB
A5	TET-CHL	A19	CEF-CXM-CAZ-SXT-GEN-CIP-CHL
A6	CEF-CXM-CHL-CIP-LVX	A20	AMK-CHL
A7	CAZ-SXT-GEN-TET	A21	CEF-CXM-CAZ-GEN
A8	SXT-TET-CHL	A21	GEN-AMK-TOB-TET-CHL
A9	CEF-CXM-SXT	A22	CEF
A10	TET	A23	CXM-AZM-TET
A11	AZM-CHL	A24	CAZ-GEN-AZM-CIP-LVX
A12	SXT-GEN-AMK	A25	CXM-TET-CHL
A13	AMK-AZM-CHL	A26	CEF
A14	CEF-CXM-SXT-CHL-CIP-IPM	A27	CXM-AZM-CHL

* Cephalothin: CEF, cefuroxime: CXM, ceftazidime: CAZ, cotrimoxazole: SXT, gentamicin: GEN, amikacin: AMK, tobramycin: TOB, azithromycin: AZM, tetracycline: TET, chloramphenicol: CHL, ciprofloxacin: CIP, levofloxacin: LVX, and imipenem: IPM.

**Table 3:** Effects of SAME Pre-treatment on serum indices of hepatotoxicity in MTX-induced hepatotoxicity in mice.

	Alanine Amino Transferase (ALT) (U/mL)	Aspartate Amino Transferase (AST) (U/mL)	Lactate Dehydrogenase (LDH) (pg/mL)
Control	41.77 ± 4.8	69.08 ± 10.2	66.82 ± 8.6
MTX	53.98 ± 6.25 ^a^	153.87 ± 12.5 ^a^	131.98 ± 9.6 ^a^
SAME 10	51.26 ± 3.8	141.78 ± 8.35	118.87 ± 10.7
SAME 20	46.92 ± 8.1	132.53 ± 7.36 ^b^	88.25 ± 5.9 ^b^
SAME 30	42.67 ± 4.5 ^bc^	78.33 ± 8.31 ^bcd^	72.78 ± 3.6 ^bcd^

Hepatotoxicity was induced by a single intraperitoneal (I.P) injection of MTX (20 mg/kg). SAME 10, 20, and 30 were given orally once daily for 5 consecutive days and a single injection of MTX on the sixth day. Data expressed as mean ± standard deviation (SD) (*n* = 8/group). Significant difference vs. ^a^ respective control, ^b^ respective MTX group, ^c^ respective SAME 10, group, ^d^ respective SAME 20 group each at *p* < 0.05.

**Table 4:** Effects of the SAME Pre-treatment on hepatic MDA level, hepatic NO content, and hepatic SOD activity in MTX-induced hepatotoxicity in mice.

	Hepatic MDA Content (nm/gm Tissue)	Hepatic NO Content (nmol/g Tissue)	Hepatic SOD Activity (U/mg Tissue)
Control	148 ± 12.2	12.7 ± 1.35	2.95 ± 0.18
MTX	230 ± 13.95 ^a^	33.2 ± 3.1 ^a^	2.1 ± 0.28 ^a^
SAME 10	210 ± 7.34	27.1 ± 2.6	2.35 ± 0.31
SAME 20	185 ± 10.4 ^b^	21.6 ± 1.92 ^b^	2.7 ± 0.21 ^b^
SAME 30	158 ± 6.51 ^bc^	14.2 ± 0.98 ^bcd^	3.05 ± 0.41 ^bcd^

Hepatotoxicity was induced by a single I.P injection of MTX (20 mg/kg). SAME 10, 20 and 30 were given orally once daily for 5 consecutive days and a single injection of MTX on the sixth day. Data expressed as mean ± SD (*n* = 8/group). Significant difference vs. ^a^ respective control, ^b^ respective MTX group, ^c^ respective SAME 10, group, ^d^ respective SAME 20 group each at *p* < 0.05.

**Table 5:** Semi-quantitative scoring shows the hepatoprotective effects of SAME.

Histological Parameters	Normal Control	MTX	SAME 10	SAME 20	SAME 30
Hepatoportal and sinusoidal congestion	-	+++	+	+	-
Hydropic degeneration	-	+++	++	+	-
Cellular necrosis	-	+++	++	+	-
Apoptosis	-	+++	+	+	-
Inflammatory cellular infiltrate	-	+++	++	+	-

- sign indicating no effect, + indicates mild effect, ++ indicates moderate effect, +++ indicates severe effect.

## Data Availability

Data are contained within the article and Supplementary Materials.

## Supplementary Materials

The following are available online at https://www.mdpi.com/article/10.3390/ph15050549/s1, Table S1. MIC values of SAME against the tested isolates Table S2. Primers used and their sequence, Figure S1. The total ion chromatograms (TIC) of *Salvinia auriculata* extract, Figure S2. NLPR3 western raw data.
