# Supplementary material for: Mechanistic Insights on the In Vitro Antibacterial Activity and In Vivo Hepatoprotective Effects of Salvinia auriculata Aubl against Methotrexate-Induced Liver Injury
Source: Pharmaceuticals (Basel). 2022 Apr 29;15(5):549. doi: 10.3390/ph15050549 (PMC9145932; doi:10.3390/ph15050549)
Supplement: Supplementary file 1 [file pharmaceuticals-15-00549-s001.zip › pharmaceuticals-1687528-supplementary.pdf]

Negative -MODE – TIC (3 Triplicate injection)

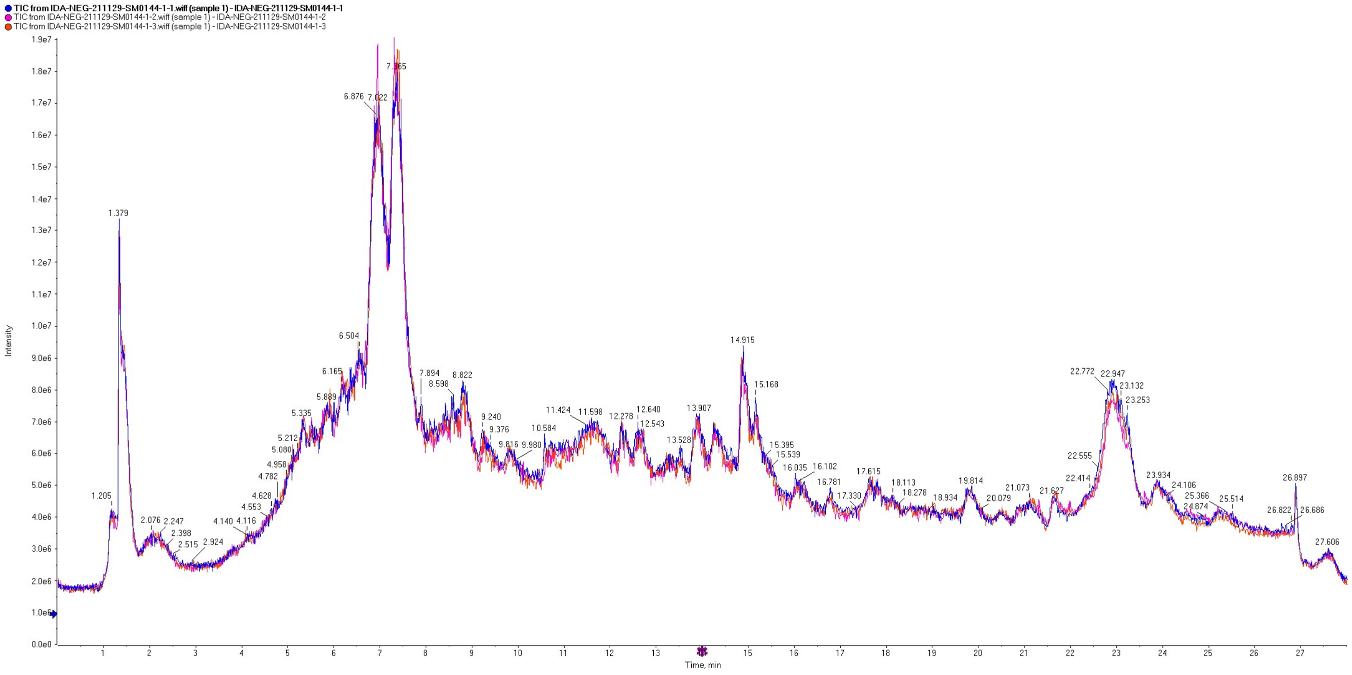

Only one sample TIC

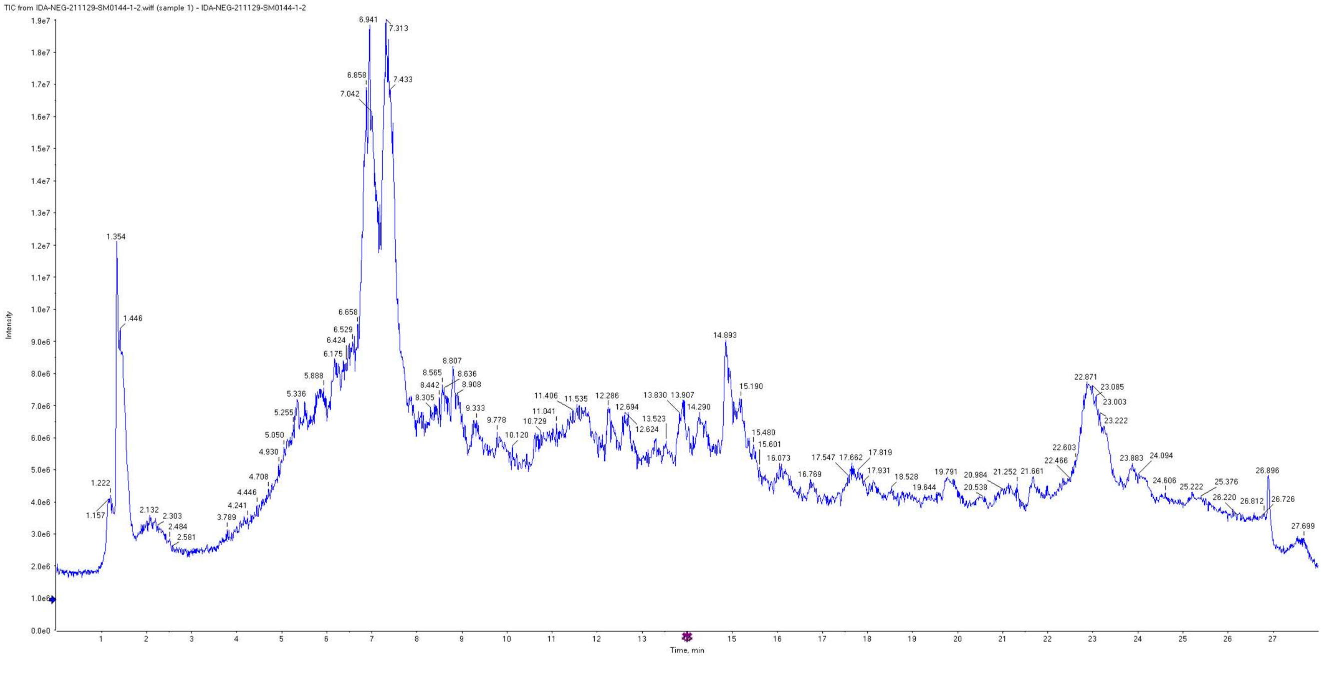

After Gaussian smooth (Smoothing width 2.0)

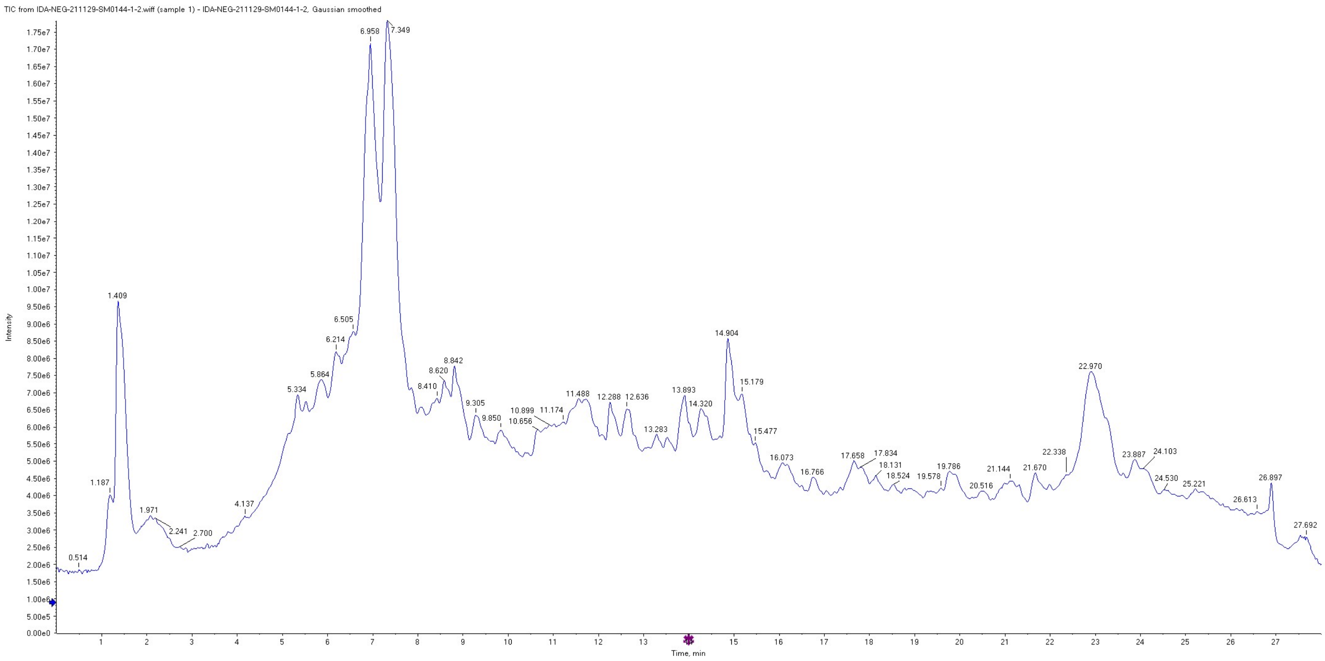

SM000: Negative -MODE – BPC

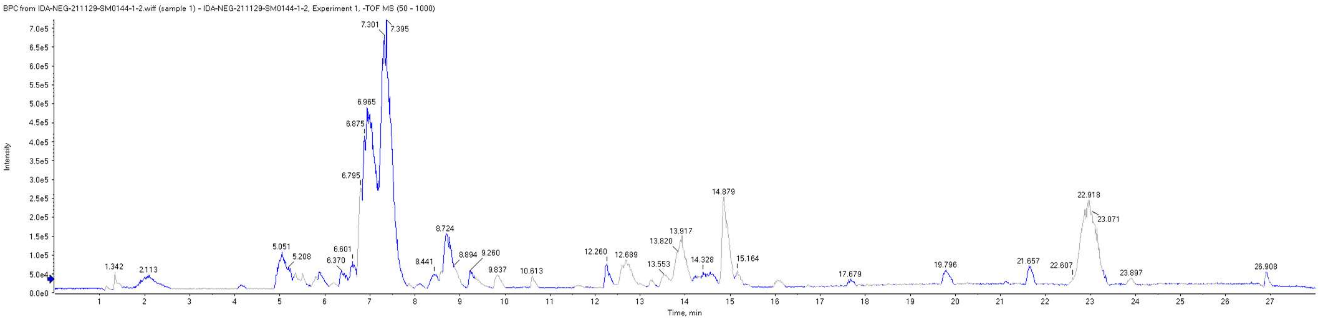

BPC -(Smoothing width 2.0)

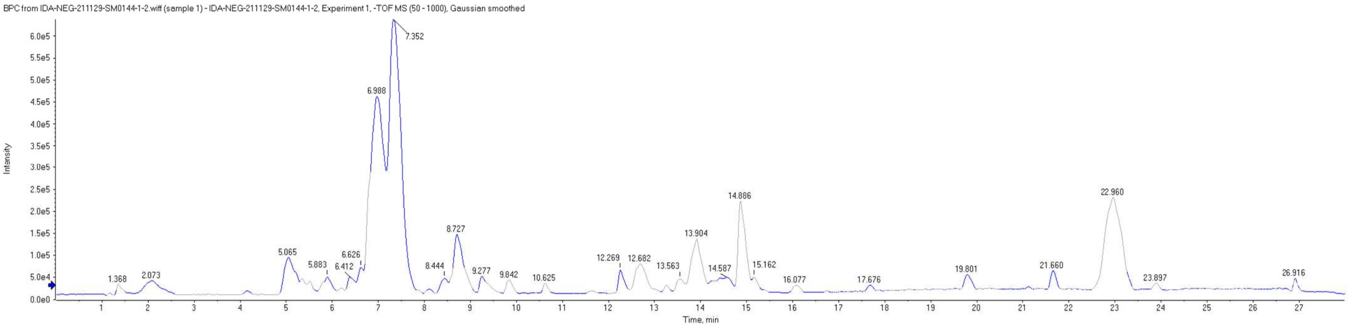

SM00144-1: Positive -MODE – TIC Duplicate injection

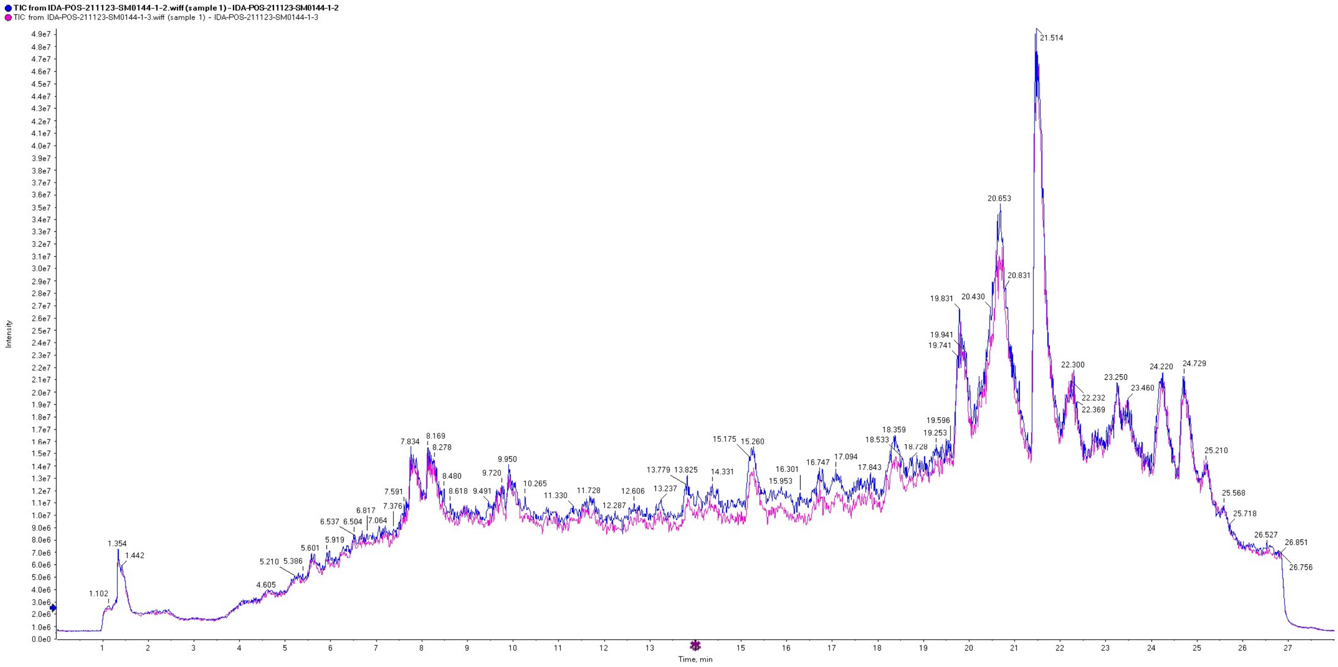

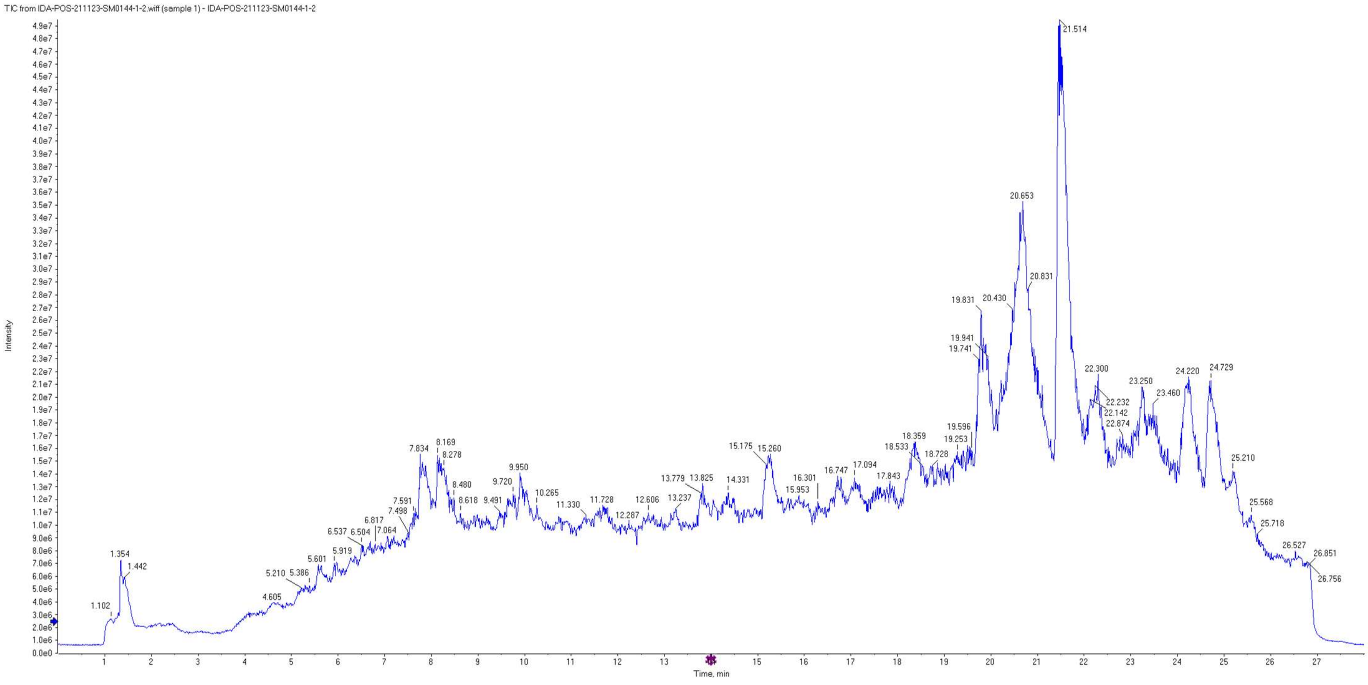

After Gaussian smooth (Smoothing width 2.0)

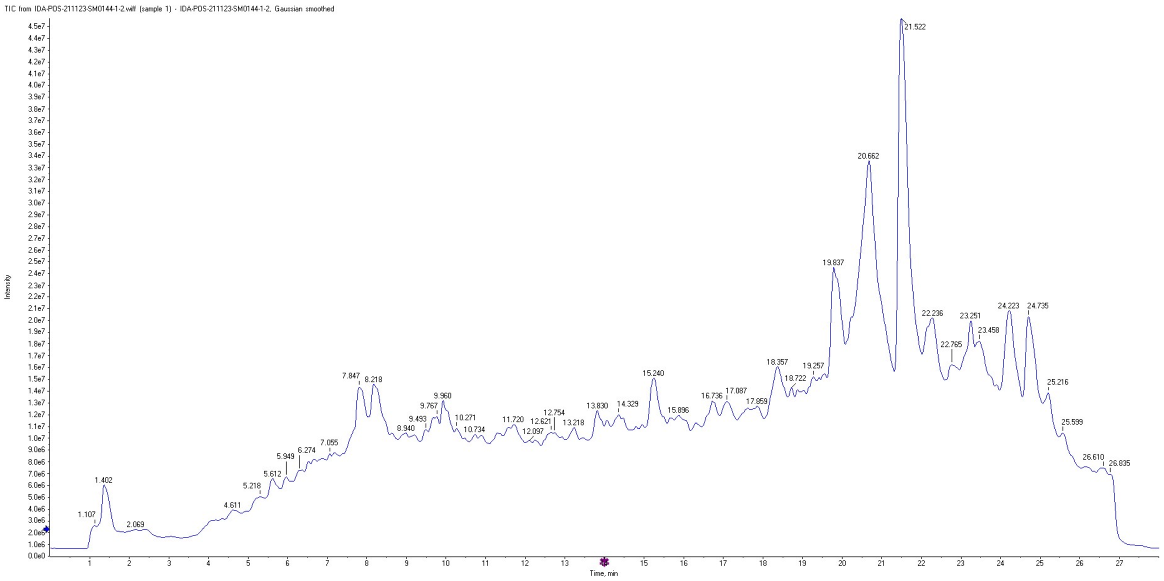

SM000: positive -MODE – BPC

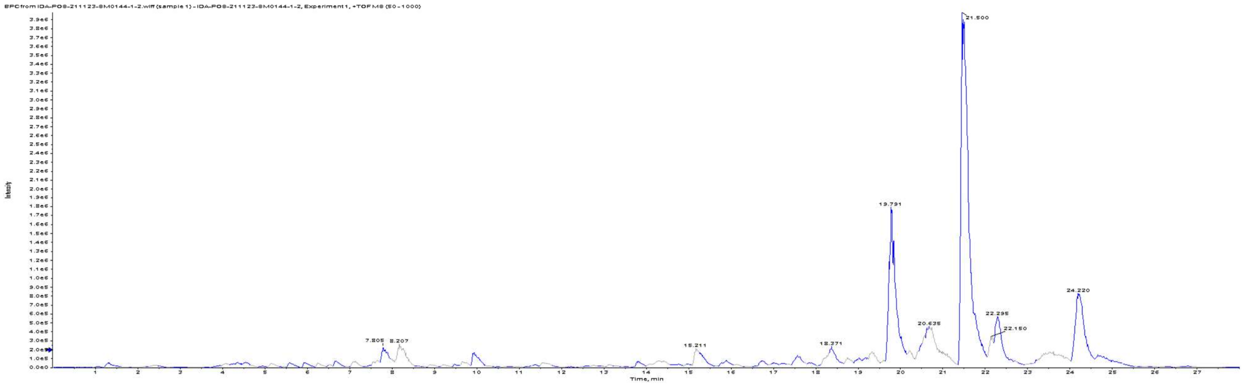

After smooth

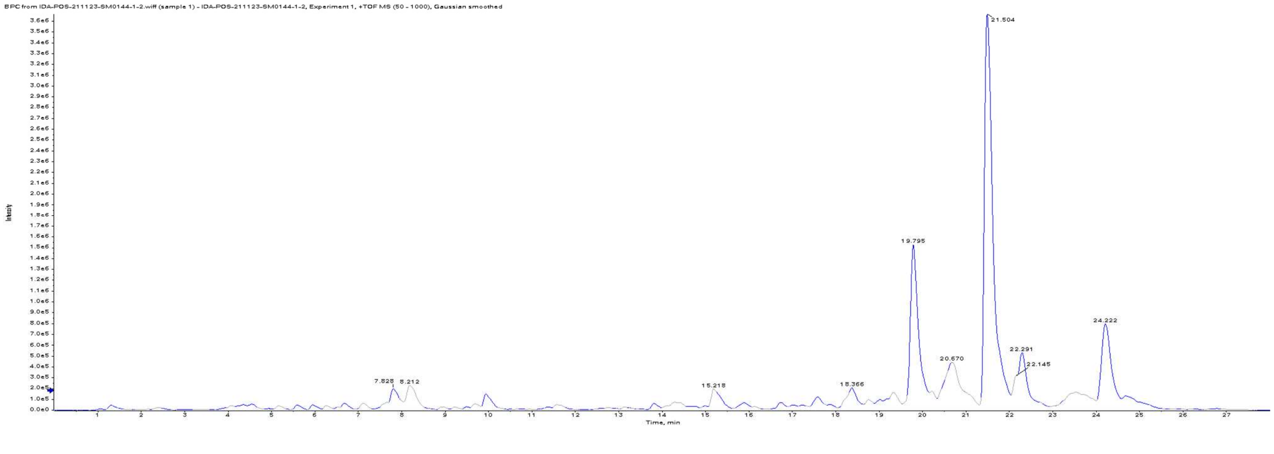

Figure S1. The total ion chromatograms (TIC) of *Salvinia auriculata* extract

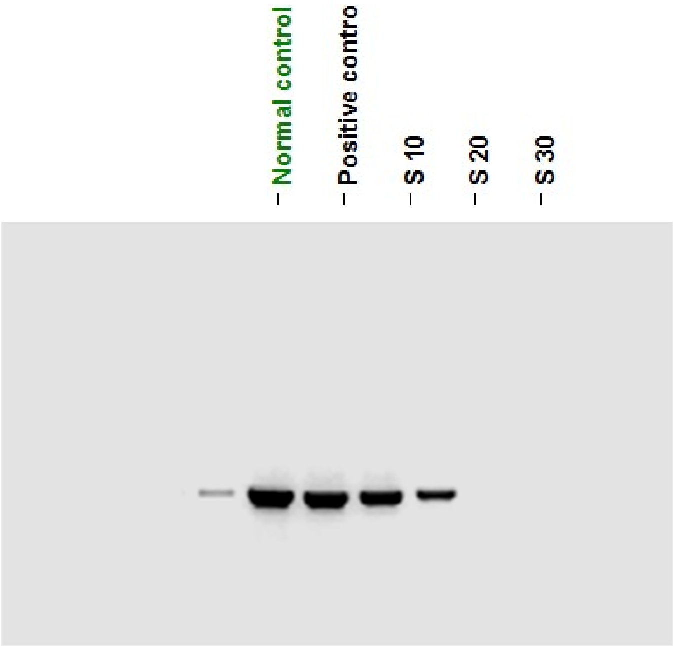

Photograph of NLPR3 antibody proein expression level for samples.

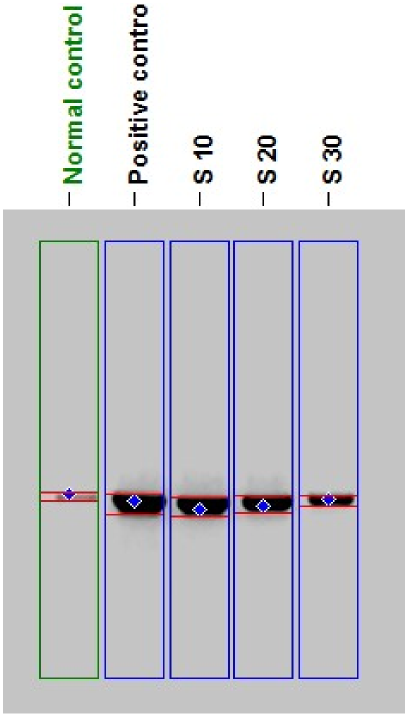

Figure of Computerized analysis of NLPR3 antibody protein expression level for samples.

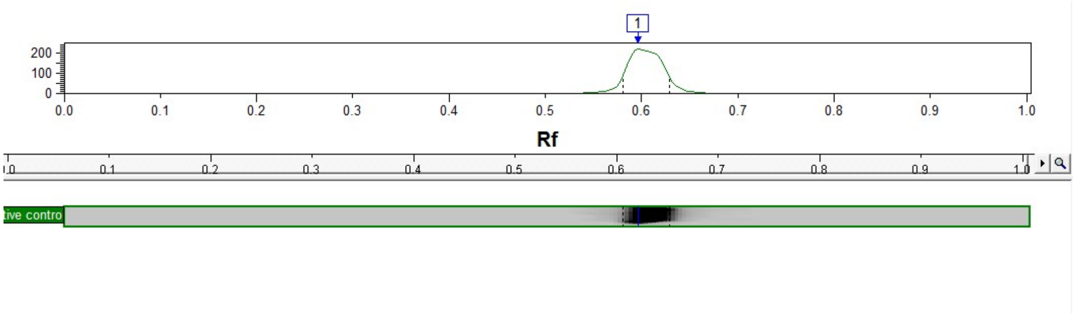

Figure of dendrogram of positive control sample.

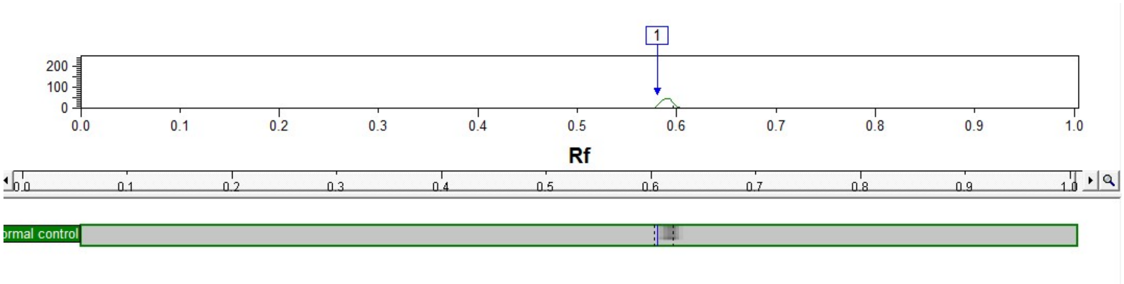

Figure of dendrogram of normal control sample.

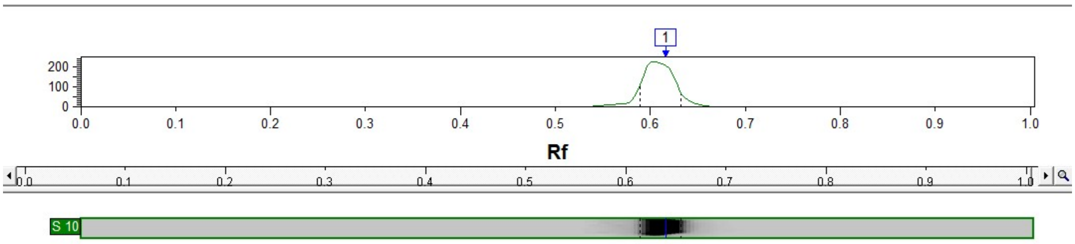

dendogram of treated sample with S 10.

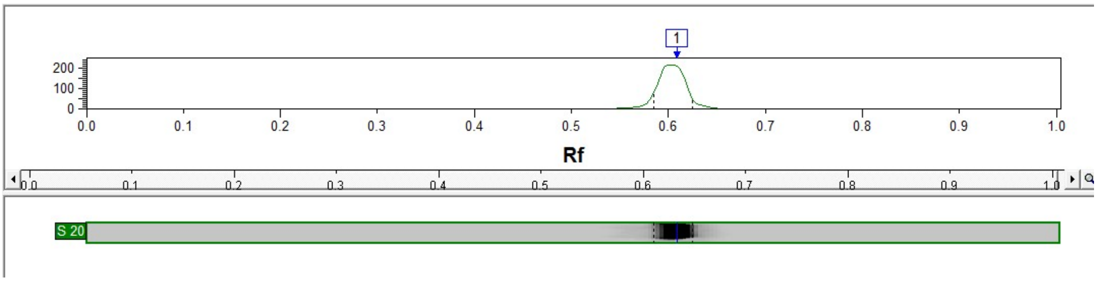

dendogram of treated sample with S 20.

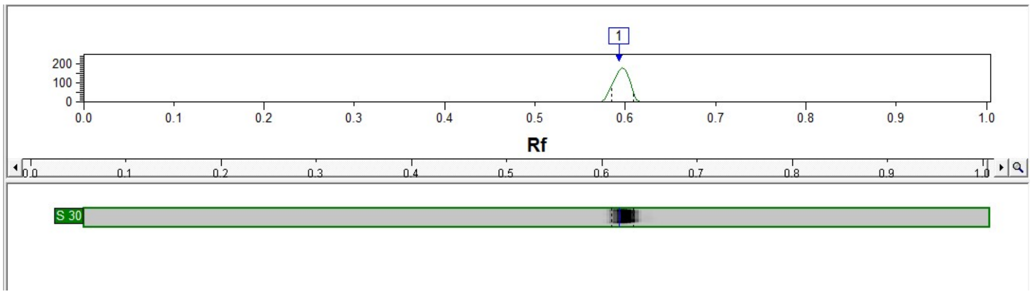

dendogram of treated sample with S 30.

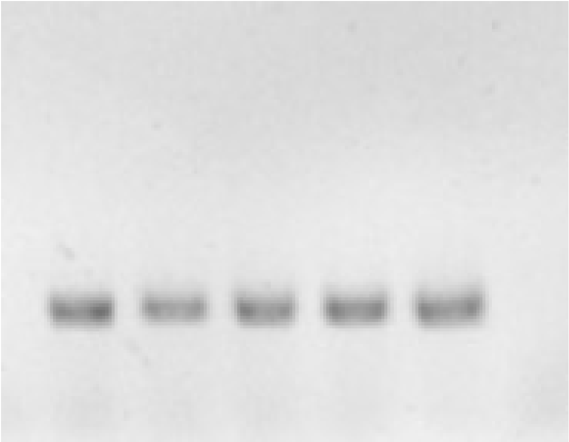

Photograph of β-actin proein expression level for samples.

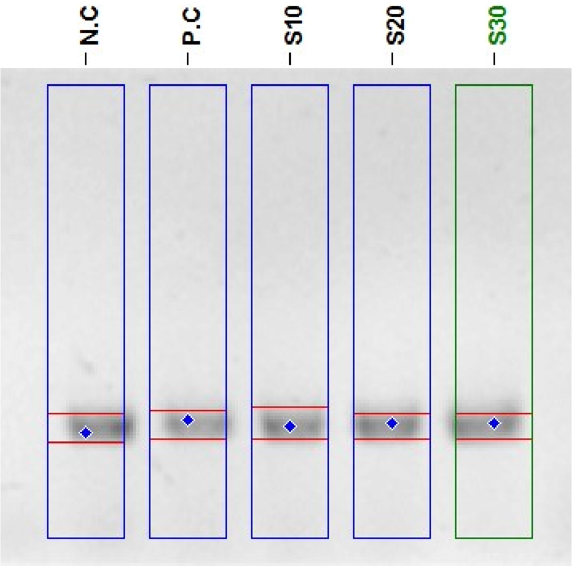

Figure of Computerized analysis of β-actin proein expression level for samples

Figure S2. NLPR3 western raw data

Table S1. MIC values of SAME against the tested isolates

Isolate code	MIC value (µg/mL)	Isolate code	MIC value (µg/mL)
**A1**	16	A15	16
**A2**	16	A16	16
**A3**	128	A17	32
**A4**	32	A18	128
**A5**	16	A19	128
**A6**	32	A20	64
**A7**	64	A21	32
**A8**	64	A22	16
**A9**	128	A23	32
**A10**	16	A24	32
**A11**	32	A25	128
**A12**	32	A26	64
**A13**	128	A27	32
**A14**	32		

Table S2. Primers used and their sequence**.**

Target Gene	Probe	Accession Number	References
**Caspase-1**	F-5′-GAA AAG GCA CGA GAC CTG TGC-3′<br>R-5′-CTT GAG GGA ACC ACT CGG TCC-3′	NM_004346	Hazman et al., 2018
**IL-1β**	ACT CCT TAG TCC TCG GCC A<br>TGG TTT CTT GTG ACC CTG AGC	NM_000576.2	Meier et al., 2019
**Nrf2**	CCATGCCTTCTTCCACGAA<br>AGGGCCCATGGATTTCAGTT	NM_031789	Espinosa et al., 2014
**IL-18**	TAA GGA TAC GGA CTA CGG CT<br>GTT GGT GGA GGT CTG AGT TTA	EF159728	Dong and Yuan 2018
**Caspase-3**	5'-TGAAGGCAAGGT GCTAAA-3'<br>5'-CTGGCTCAAACCACATTCTC-3'	NM_001284409.1	LUAN et al., 2007
**GADPH**	5′-AGAAGG CTGGGGCTCATTTG-3′<br>5′-AGGGGCCAT CCACAGTCTTC-3′	NM_008084	Jing-Jing et al., 2012
